# MicroFPGA: An affordable FPGA platform for microscope control

**DOI:** 10.1016/j.ohx.2023.e00407

**Published:** 2023-02-24

**Authors:** Joran Deschamps, Christian Kieser, Philipp Hoess, Takahiro Deguchi, Jonas Ries

**Affiliations:** a Computational Biology Center, Fondazione Human Technopole, Milan, Italy; b Cell Biology and Biophysics Unit, European Molecular Biology Laboratory, Heidelberg, Germany; c Electronics Workshop, European Molecular Biology Laboratory, Heidelberg, Germany

**Keywords:** FPGA, Microscopy, Electronics, Triggering, Synchronization, Automation, **ACB**, analog conversion board, **AOM**, acousto-optic modulator, **AOTF**, acousto-optic tunable filter, **AOTF-CB**, AOTF conversion board, **BOM**, bill of materials, **EMCCD**, electron multiplying charge-coupled device, **FPGA**, field-programmable gate array, **GND**, ground, **HDL**, hardware description language, **I/O**, input/output, **PWM**, pulse-width modulation, **TTL**, transistor-transistor logic, **SCB**, signal conversion board, **(s) CMOS**, (scientific) complementary metal–oxide–semiconductor, **SDB**, servo distribution board

## Abstract

Modern microscopy relies increasingly on microscope automation to improve throughput, ensure reproducibility or observe rare events. Automation requires computer control of the important elements of the microscope. Furthermore, optical elements that are usually fixed or manually movable can be placed on electronically-controllable elements. In most cases, a central electronics board is necessary to generate the control signals they require and to communicate with the computer. For such tasks, Arduino microcontrollers are widely used due to their low cost and programming entry barrier. However, they are limiting in their performance for applications that require high-speed or multiple parallel processes. Field programmable gate arrays (FPGA) are the perfect technology for high-speed microscope control, as they are capable of processing signals in parallel and with high temporal precision. While plummeting prices made the technology available to consumers, a major hurdle remaining is the complex languages used to configure them. In this work, we used an affordable FPGA, delivered with an open-source and friendly-to-use programming language, to create a versatile microscope control platform called MicroFPGA. It is capable of synchronously triggering cameras and multiple lasers following complex patterns, as well as generating various signals used to control microscope elements such as filter wheels, servomotor stages, flip-mirrors, laser power or acousto-optic modulators. MicroFPGA is open-source and we provide online Micro-Manager, Java, Python and LabVIEW libraries, together with blueprints and tutorials.

## Graphical abstract

**Figure:**
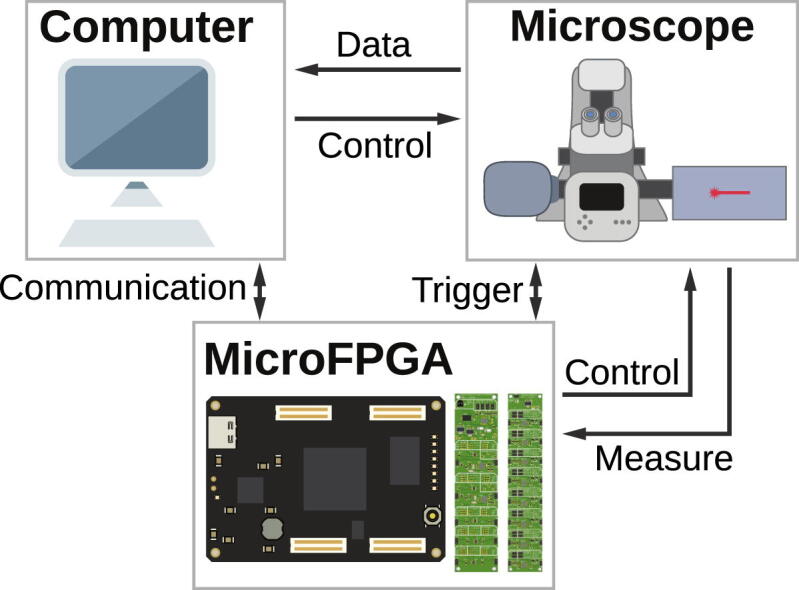

**Specifications table**

**Table:** 

**Hardware name**	MicroFPGA
**Subject area**	• Engineering and Material Science • Biological Sciences • Educational tools and open source alternatives to existing infrastructure
**Hardware type**	• Imaging tools • Electrical engineering and computer science
**Closest commercial analog**	Triggerscope (Advanced Research Consulting)
**Open source license**	MIT, BSD-3 Clause
**Cost of hardware**	• ≈ €75–360 without optional electronics • ≈ €1200 for the full electronics box (self-assembled from ready-soldered boards)
**Source file repository**	Zenodo (design files), Github (source code)

## 1 Hardware in context

Microscope automation is a promising avenue for biological studies. Not only does it save time for researchers, but it also substantially increases the throughput of the microscopes, improving statistical power or enabling the observation of rare events [1]. In order to automate microscopes, all elements usually moved by hand must become computer-controlled. This often equates to mounting optics on motorized stages or servomotors, adding electronically-controlled flip-mirrors and shutters, and using electronics to synchronize devices such as cameras and lasers. Furthermore, automation enables the implementation of new modalities on the microscope, thus increasing the flexibility of the imaging. For instance, in fluorescence microscopy, lasers are often triggered using a camera signal in order to avoid unnecessary bleaching of the sample outside of the camera acquisition time frame. Adding a layer of signal processing in between the camera and the lasers enables more complex triggering patterns, such as fast pulsing or interleaved illumination.

Often, a central electronic board communicates with the computer and delivers the control signals to the various elements in order to move them into position. Typical signals include transistor-transistor logic (TTL), pulse-width modulation (PWM) or analog signals. Furthermore, reading out analog signals is a useful addition to monitor the microscope state, such as its temperature or laser power. Because instruments are subject to change, reprogrammable electronics are almost always preferred to application specific integrated circuits, as the cost of a new circuit production and long design-testing cycles are not suitable for research projects.

In that respect, Arduino microcontrollers have the advantage of being affordable, easy to program, open-source and benefit from a large community. They are therefore at the heart of many open-source microscopy projects [2], [3], [4], [5], [6], [7]. An intrinsic limitation of microcontrollers is that they are most often based on a single CPU, and therefore cannot perform multiple tasks in parallel. Processes, such as generating signals to drive servomotors, compete in CPU resources, limiting temporal resolution in signal processing. Careful optimization and use of interrupts can help perform complex tasks without perceptible delays. However, this approach does not scale well with an increasing number of processes and high-speed signal generation. Nonetheless, Arduino boards are sufficient for a very wide range of applications, in particular when high-speed processing is not crucial.

When the tasks at hand are too complex for microcontrollers, another solution is the use of field-programmable gate arrays (FPGA). FPGAs are integrated circuits consisting of millions of logic blocks that can be configured to perform the required tasks. Because independent tasks are broken down into different logic block ensembles, signals are processed concurrently. The number of processes that can run independently is then only limited by their complexity and the size of the FPGA. Thus, FPGAs are preferable to microcontrollers for applications requiring fast processing and precise timing. They are, however, considerably more complex to program. They typically require hardware description language (HDL) and advanced optimization tools, such as those provided by their manufacturers, limiting their use to specialists. Nonetheless, FPGAs have become more accessible, with more online resources available and plummeting prices [8]. National Instruments (NI) offers several FPGA modules compatible with its commercial and proprietary platform, LabVIEW. These modules have found important applications in advanced optical microscopy [9], [10], [11], [12], [13], [14], synchronizing complex microscopes for high-speed imaging or processing. The FPGA modules and software are aimed at high-end applications, with an unreasonable cost for every-day electronics control and straightforward signal processing tasks.

Here, we present MicroFPGA, a low-cost platform for electronic control of microscope devices aimed at providing a toolbox for automating microscopes. MicroFPGA is based on affordable FPGAs (Au+ or Cu, Alchitry), which rely solely on open-source or freely available software. The FPGAs are delivered with their own development environment (AlchitryLabs, Alchitry) and can be configured with a comparatively friendly HDL language: Lucid [15]. MicroFPGA includes a powerful camera-laser synchronization module and laser triggering system, and can generate widely used electronic signals, such as TTL, PWM or servo control signals. In addition, the Au+ FPGA boards can read multiple analog signals. We also provide blueprints for complementary electronic boards, allowing voltage conversion and the generation of analog output signals from the PWM outputs. MicroFPGA can be controlled via the open-source microscope control software Micro-Manager [16], or via our Python and Java libraries. Although LabVIEW is costly and not open-source, it is widely used in the microscopy community; we therefore also provide a compatible communication library, in order to help narrow the gap between commercial instruments and open-source initiatives [17], [18], [19]. All source code as well as user guide and programming tutorials is available online [20], [21], [22].

## 2 Hardware description

**Fig. 1:**
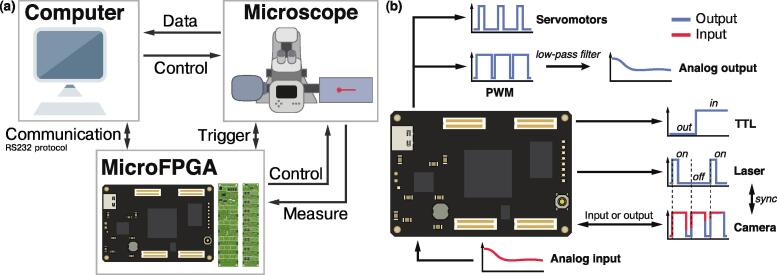
MicroFPGA overview. (a) MicroFPGA communicates with the computer over USB (RS232 protocol) and allows controlling or triggering elements on a microscope, as well as measuring signals. (b): Overview of the input and output signals of MicroFPGA.

MicroFPGA aims at expanding the flexibility of a microscope by enabling computer control of existing or new microscope elements. As such, it serves as intermediary between the computer and the microscope in order to control these elements or measure signals from the microscope (see Fig. 1a). MicroFPGA can generate several types of signals commonly used to control devices in microscopy (see Fig. 1b) and is based on Alchitry FPGAs (Au+ and Cu). The platform consists of the source code required to configure the FPGA boards, as well as libraries to communicate with the FPGA from open-source software (Micro-Manager) or languages (Python and Java). Additionally, we released a communication library compatible with LabVIEW. Communication is performed via RS232 serial communication over the FPGA board’s USB port. We also supply optional electronic boards for analog output and voltage conversion, and blueprints for integrating them into a packaged electronics box.

By default, MicroFPGA provides 6 different modules (see Table 1): camera synchronization (1 input and 1 output channel), laser trigger (8 output channels), analog read-out (8 input channels), PWM (5 output channels), servomotors (7 output channels) and TTL (4 output channels). The camera synchronization and laser trigger modules enable complex synchronization between a camera and multiple lasers. The camera can either trigger or be triggered by the FPGA. The lasers are triggered independently following intricate patterns determined by a set of parameters (see Table 1) and with high temporal precision. The other modules generate common signals used to control devices. In particular, the PWM channels can be low-pass filtered to generate analog output signals, and even synchronize them with a camera, using the optional electronics. The number of channels for each module can be changed (except the analog input) by modifying the FPGA source code. We provide more details on each signal type in **7. Validation and characterization**.

**Table 1:** MicroFPGA inputs/outputs.

**Signal**	**I/O**	**Parameters**	**Board**	**# channels**
Camera	input	–	Au+ / Cu	1
Camera	output	mode, start, pulse, delay, exposure, read-out	Au+ / Cu	1
Laser	output	mode, duration, sequence	Au+ / Cu	8
Analog	input	state	Au+	8 (fixed)
PWM	output	state	Au+ / Cu	5
Servo	output	state	Au+ / Cu	7
TTL	output	state	Au+ / Cu	4

MicroFPGA thus offers a variety of signals ”out-of-the-box”, allowing to control many aspects of a microscope, such as synchronizing camera and lasers, moving optical elements using servomotors, controlling AOM/AOTF or flip-mirrors, or reading out sensors. It is not limited to the control of custom-built microscopes and can be used with any set of devices that are physically accessible to users. The most obvious application with commercial microscope is to place MicroFPGA between camera and lasers to enable complex triggering.

Central electronic controllers, tasked with moving elements, synchronizing devices and reading sensor values, are most commonly based on microcontrollers, such as Arduino. FPGAs have a clear advantage over microcontrollers in their capacity of processing signals in a parallel fashion and at higher speed. Additionally, increasing the number of tasks carried out by an FPGA does not require timing optimization as long as the tasks are independent and the FPGA has enough free logic cells. The same situation is not true for microcontrollers, where any new task requires careful optimization in order to not cause delays to other tasks, when it is possible at all. Typically, triggering several lasers in parallel at microsecond scale is a difficult task to achieve with an Arduino, while it poses no hurdle to an FPGA. As opposed to Arduino-based systems, MicroFPGA can trigger all connected lasers independently following complex sequences, with less than 100ns delays and with μs pulse resolution, regardless of how many laser outputs are used.

A commercial alternative is TriggerScope (Advanced Research Consulting), which features numerous digital and analog outputs synchronized with four digital inputs that is compatible with Micro-Manager. Triggerscope is based on a Teensy microcontroller (PJRC) with a fast processor, mitigating delays commonly observed with microcontrollers while synchronizing multiple output channels. It allows controlling a wide array of devices by offering a choice of output voltage ranges and has the possibility to run pre-loaded sequences, a feature particularly useful for enabling complex synchronizations between camera and downstream devices such as stages. Other types of signals, such as servomotor signals or specific synchronization patterns, need to be implemented on a custom basis with the manufacturer. As opposed to TriggerScope, MicroFPGA can only synchronize digital and analog signals with a single channel. However, MicroFPGA has superior synchronization speed thanks to its FPGA, and its source code can be easily modified for applications requiring different signals or synchronization patterns. Furthermore, it also generates servomotor signals and can read analog signals without any modification to the code.

NI offers a variety of boards with analog and digital I/O, including a FPGA module. These boards are in wide use, especially in advanced microscopes. However, they require users to implement their own FPGA configuration using LabVIEW (NI), NI’s proprietary platform. Software and hardware together amount to several thousands of euros more than MicroFPGA, especially when including the NI FPGA module. For comparatively simple tasks such as those solved by MicroFPGA, the main advantage of National Instrument’s FPGA module is the relative low entry barrier of LabVIEW coding. FPGAs are usually configured using HDL languages, which have a steep learning curve. Since MicroFPGA is based on the FPGA boards from Alchitry, it was written in Lucid, a more user-friendly HDL language developed by the manufacturer. In addition, Alchitry provides a continuously expanding set of tutorials on their website. Thus, users wanting to modify MicroFPGA can draw inspiration from these tutorials and modify the source code to fit their applications. To this end, we also uploaded to the MicroFPGA repositories brief tutorials covering a range of useful modifications of the code, such as changing the number of signals available in MicroFPGA, remapping the output pins, creating signals to control more exotic servomotors or changing the parameters’ dynamic range.

The key features of MicroFPGA are:

• Fast and flexible triggering of multiple lasers
• Camera triggering and synchronization with lasers
• Electronic control of a wide array of microscope elements
• Analog input and output signals
• Compatible with Micro-Manager, Python, Java and LabVIEW

## 3 Design files summary

The design files concern the optional electronics developed for MicroFPGA, packaged within a 3D printed box. The electronics box is tailored to our use of the platform and corresponds to MicroFPGA default signals.

**Table:** 

**Design filename**	**File type**	**Open source license**	**Location of the file**
Analog conversion board	Altium project, Gerber	MIT	Zenodo
Signal conversion board	Altium project, Gerber	MIT	Zenodo
Box panel 1 (device connections)	Altium project, Gerber	MIT	Zenodo
Box panel 2 (device connections)	Altium project, Gerber	MIT	Zenodo
Box panel 3 (voltage source)	Altium project, Gerber	MIT	Zenodo
FPGA shield	Altium project, Gerber	MIT	Zenodo
Electronics box	STEP, EASM	MIT	Zenodo
Servo distribution board	Altium project, Gerber	MIT	Zenodo
AOTF conversion board	Altium project, Gerber	MIT	Zenodo

*Analog conversion board (ACB)* is a complementary board used to convert analog signals from 0–5 V or 0–10 V to 0–1 V, the range expected by the Au+ FPGA analog input.

*Signal conversion board (SCB)* is a complementary board capable of bidirectionally scaling digital voltages between 0–3.3 and 0–5 V. In addition, the board features two unidirectional low-pass filters that allow converting PWM outputs to analog signals.

*Box panel 1, 2 and 3* are the side panels of the electronics box. They feature standard connectors to facilitate wiring the electronics box to devices. Box panel 3 supplies different power to the other boards.

*FPGA shield* is a custom FPGA shield to be stacked on top of a Br shield (Alchitry) to simplify connecting the electronics box side panels to the FPGA pins.

*Electronics box* contains the design files for the 3D printed housing.

*Servo distribution board (SDB)* is a board that simplifies connecting multiple servomotors to the electronics box.

*AOTF conversion board (AOTF-CB)* generates multiple analog signals that can be switched on and off by input TTL signals, the analog signals are themselves generated by low-pass filtering PWM inputs. This board allows synchronizing analog signals with a camera using MicroFPGA laser triggers as TTL inputs.

## 4 Bill of materials summary

MicroFPGA can be used by simply buying an Alchitry FPGA (Cu or Au+) and a Br shield. The optional electronics is custom-made and can be ordered with PCB (printed circuit board) manufacturers with all components already soldered. The bill of materials for each optional electronic element can be found with the design files [22]. The costs for the custom boards will depend on the manufacturers and can vary with the number of boards ordered or general price fluctuations, the costs indicated in the table are therefore rough estimates only. The overall price can be decreased by ordering the PCB only and soldering the components oneself. The ACB and SCB were ordered assembled from a manufacturer (Sigmann Elektronik) with ordering numbers 2020–30170 and reference ”Analog Conversion 8 Channels” and ”Signal Conversion 2 + 3 Channels”. The enclosing box is composed of a 3D printed box and a machined lid, the cost of which will depend on access to a mechanical workshop. Finally, cables are necessary to connect devices to the FPGA, through the optional electronics or directly onto the Br shield, and depend on the devices themselves.

**Table:** 

**Component**	**Number**	**Total cost**	**Source**	**Material type**
FPGA	1	€47.32 (Cu) or €337.16 (Au+)	SparkFun	semi-conductor

Breakout shield	1	€14.20	SparkFun	semi-conductor
*ACB*	1	≈ €200	*Sigmann Elektronik*	semi-conductor
*SCB*	1	≈ €130	*Sigmann Elektronik*	semi-conductor
*FPGA shield*	1	≈ €100	*any PCB manufacturer*	semi-conductor
*Box panel 1*	1	≈ €100	*any PCB manufacturer*	semi-conductor
*Box panel 2*	1	≈ €100	*any PCB manufacturer*	semi-conductor
*Box panel 3*	1	≈ €100	*any PCB manufacturer*	semi-conductor
*SDB*	1	≈ €50	*any PCB manufacturer*	semi-conductor
*AOTF-CB*	1	≈ €150	*any PCB manufacturer*	semi-conductor
*Cables and screws*	-	≈ €50	–	metal, plastic
*Power supply*	1	≈ €30	–	plastic
*Box housing*	1	≈ –	*Self-printed*	plastic

The elements in italic are optional and depend on the application (see **”How to choose the build level”** in the next section).

## 5 Build instructions

### 5.1 General safety notice

The FPGA features 1.8 V and 3.3 V digital pins (Cu and Au+) and an ADC measuring analog voltages up to 1 V (Au+). The 1.8 V pins are not used in MicroFPGA. In order to prevent damaging the board, never input voltages higher than these ranges to the FPGA. Test input voltages before wiring cables to the FPGA pins. All usual safety precautions should be respected when working with electronics or while soldering.

### 5.2 General assembly notice

The necessity to build the optional electronics depends on user applications. We advise assembling a list of the devices meant to be controlled or synchronized with MicroFPGA. For each device, check the data sheet or contact the vendor to determine the type of signal required to control the device along with the voltage requirements. This will determine whether to build the electronics box. We describe in this manuscript two different build levels: (i) MicroFPGA without optional electronics, and (ii) MicroFPGA with the complete electronics box. The complete electronics box follows an example implemented on our microscope and the number of channels can be altered to fit the need of your application. In order to build the complete electronics box, an oscilloscope, a function generator and a variable power supply with current display and current protection are recommended to verify the integrity of the electronic boards along the way.

### 5.3 How to choose the build level

The first step is to determine which FPGA to use between Au+ and Cu boards. The Au+ features a much larger FPGA and the capacity to read analog voltages, albeit at a higher price. Unless users intend to extend MicroFPGA with more complex tasks or increase the number of signals, the size of the on-board FPGA should not matter. Therefore, the main criterion to choose between the two FPGA boards is whether reading analog voltages is necessary for the particular application.

Then, users should determine whether to build the complementary electronics. The ACB board is used to convert analog voltages to the 0–1 V range before the Au+ FPGA. As stated above, for applications that do not require reading analog voltages, users should opt for the less expensive Cu FPGA and avoid building the ACB. The SCB has multiple functions: converting FPGA outputs to the 0–5 V range, converting inputs to the FPGA to 0–3.3 V and generate analog voltages from the FPGA PWM channels. In general, the 3.3 V logic of the FPGA digital outputs can interface with 5 V devices, meaning that the FPGA outputs can be used with downstream devices without the SCB. A typical use case for the SCB is to downscale a camera trigger input to 3.3 V before the FPGA. Most (s) CMOS and some EMCCD directly output 3.3 V signals, in which case this functionality is not used. Finally, the SCB allows generating analog voltage outputs to control devices such as AOM/AOTF or stages.

If none of the complementary electronics (ACB and SCB) are necessary, then MicroFPGA can be used simply with the bare FPGA board and the break-out shield (Br). On the contrary, if one or both of the electronic boards are required, we advise building the full electronics box. The box ensures electrical stability, as well as clear identification of the outside ports to which each device should be connected. However, it constraints the number of output for each type of signal to a fixed number and might not fit all applications. Users should then adapt the box electronics, either by changing the internal wiring or by redesigning parts of it.

Finally, two additional boards are included in the project: SDB and AOTF-CB. The SDB is used together with the electronics box to distribute the servo signals generated by the FPGA. The AOTF-CB is similar to the SCB in the sense that it also generates analog signals by low-pass filtering PWM. However, it includes the possibility to quickly turn on or off the signals using TTL inputs. Thus, it allows synchronizing analog signals with a camera, for instance when controlling a laser output with an AOTF.

### 5.4 MicroFPGA without optional electronics

#### 5.4.1 Configuring MicroFPGA

1. Download and install AlchitryLabs from Alchitry’s website. This procedure also installs AlchitryLoader.
2. Download the relevant FPGA configuration (*.bin* file) from the release page of the MicroFPGA source code repository [20].
3. Connect the FPGA to the computer and upload the configuration to the flash memory of the FPGA using AlchitryLoader.

Alternatively, the source code can also be built locally before uploading it to the FPGA. This requires installing iCEcube2 (Lattice semiconductors) or Vivado WebPACK (Xilinx) for the Cu and Au+, respectively. Both of these software require registering with the vendor to obtain a free license. Once the tool-chains are installed, the source code [20] can be modified, built and uploaded to the FPGA using AlchitryLabs.

#### 5.4.2 Controlling MicroFPGA

MicroFPGA is included in Micro-Manager and can be used by downloading a recent version and loading the correct device adapter (with baud rate 57600). Each parameter shown in Table 1 can be changed for each channel individually through Micro-Manager’s interface (device property browser) or mapped to the graphical elements of a plugin [23]. Refer to Micro-Manager’s wiki for further instructions on how to use the software [24]. Alternatively, MicroFPGA can be controlled from Python, Java and LabVIEW using the relevant libraries [20]. The Python package and Java library come with multiple examples, and the Python package is available on PyPi.

#### 5.4.3 Soldering the breakout shield

**Fig. 2:**
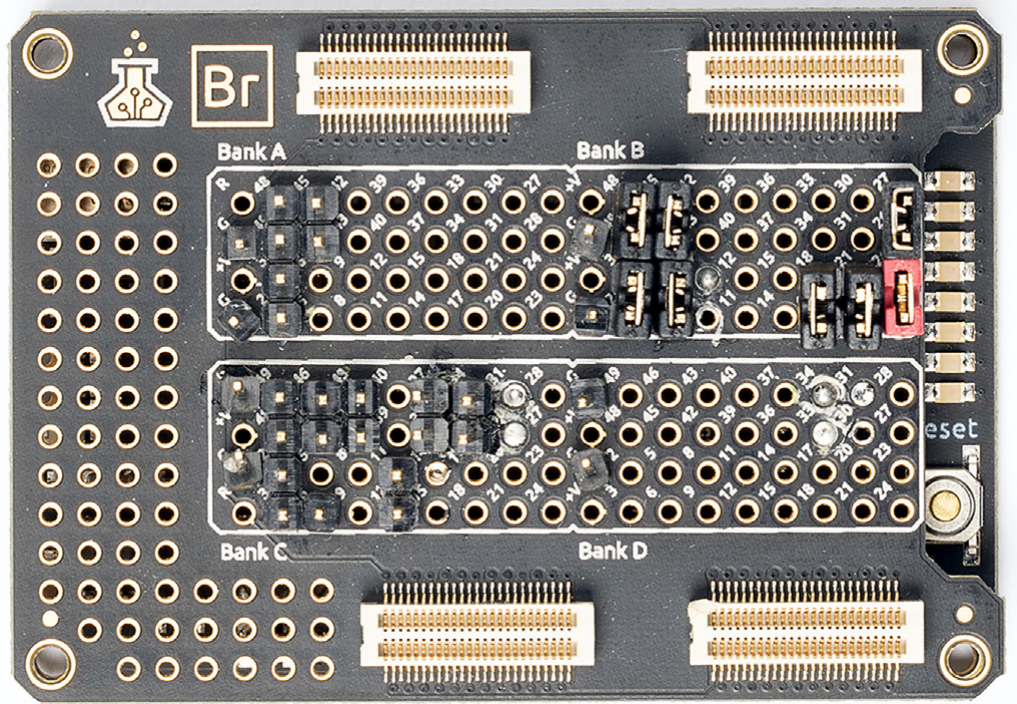
Br shield (Alchitry) with some pins soldered. Photography credit: EMBL Photolab - Stuart Ingham.

The Br shield allows accessing the different FPGA pins more easily. If you intend to only use the FPGA and the Br shield, without the full electronics box, then soldering male pins (2.54mm THT) to the Br board is the easiest and most convenient way to wire the FPGA with other devices (see an example in Fig. 2).

1. List the inputs and outputs that you intend to use.
2. Refer to Table 3, the online documentation or the source code to locate the I/O on the Br shield. Note that analog inputs of the Au+ FPGA are polar and have both a positive and a negative pin.
3. Solder male-male pins on each relevant hole in the Br shield, or entire pin headers for better stability.
4. Split GND pins by soldering multiple cables together on GND pins of the Br.
5. Use a multimeter to verify that all FPGA outputs are correctly soldered.
6. To verify that the pins are soldered on the correct holes, change the value of MicroFPGA’s outputs using one of the libraries (Micro-Manager, Python, Java, LabVIEW) and validate the signals on an oscilloscope.
7. For the Au+ FPGA, use a variable voltage source as input to the analog input pins and use the software of your choice to read out the values. Note that these pins work in pairs of positive and negative pins. **Do not input more than 1** **V to the FPGA.**
8. Use a function generator to generate a square TTL signal (0–3.3 V) and input it to the *camera in* pin. Set the FPGA to passive synchronization mode with lasers in follow mode (mode  = 4) and sequences to their maximum value (65 535), and use an oscilloscope to verify the laser triggering by displaying the function generator and the FPGA laser output signals together.

#### 5.4.4 Connecting to devices

The connection to the devices requires compatible cables on the device side (BNC, SMA, SMB etc.) and female pin adapters to connect to the Br shield. Servomotors need an additional power supply to function. We advise using an external power supply to avoid overloading the FPGA. Make sure to connect the power supply GND to the FPGA.

### 5.5 MicroFPGA with electronics box

**Fig. 3:**
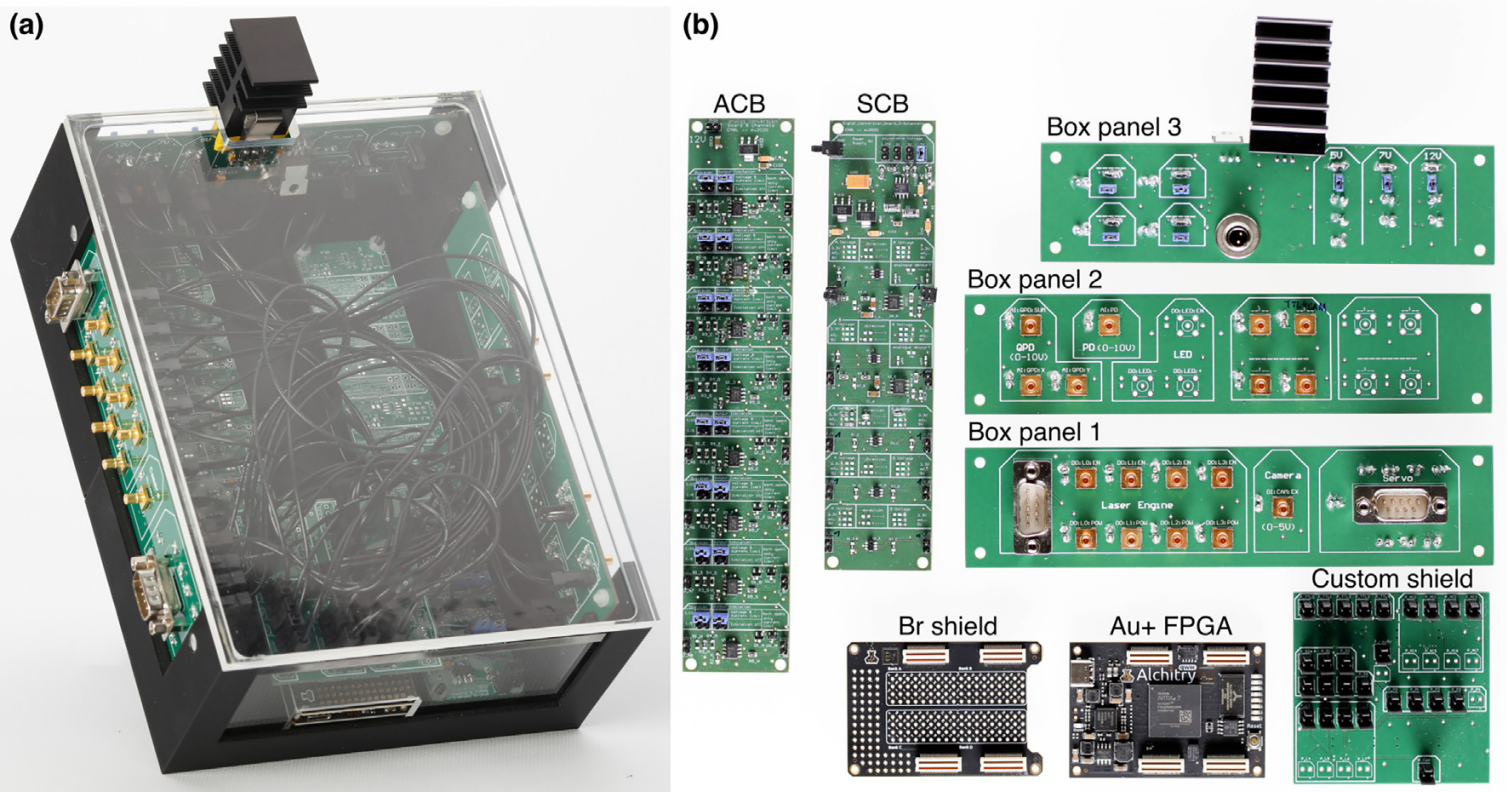
Overview of the electronics box. (a) Complete electronics box. (b) Overview of the different boards used inside the electronics box. Photography credit: EMBL Photolab – Stuart Ingham.

Our microscopes make use of both complementary electronics (ACB and SCB). In order to avoid hand-soldered cables and ensure electronic stability, we designed a complete electronics box (see Fig. 3a). The housing is 3D printed and accommodates 3 electronic boards as side panels. The function of the side panels is to relay the signals from the FPGA and complementary boards to other devices, as well as generating the correct voltages for the different boards. The advantage of this build is that connecting devices to the box does not require opening the box: all I/O are accessible on the outside of the box via standard connectors (D-SUB9 and SMB). An overview of the different electronic elements of the box is shown in Fig. 3b. Finally, the servomotor D-SUB9 connector already includes a 5 V power supply pin, allowing to power servomotors via the SDB. The bill of materials for each element can be found in their respective project folder [20], [22]. We advise ordering all the custom boards with the components already soldered. Expert users or those having access to an electronics workshop can potentially decrease the total price of the build by ordering PCB and components independently.

**Fig. 4:**
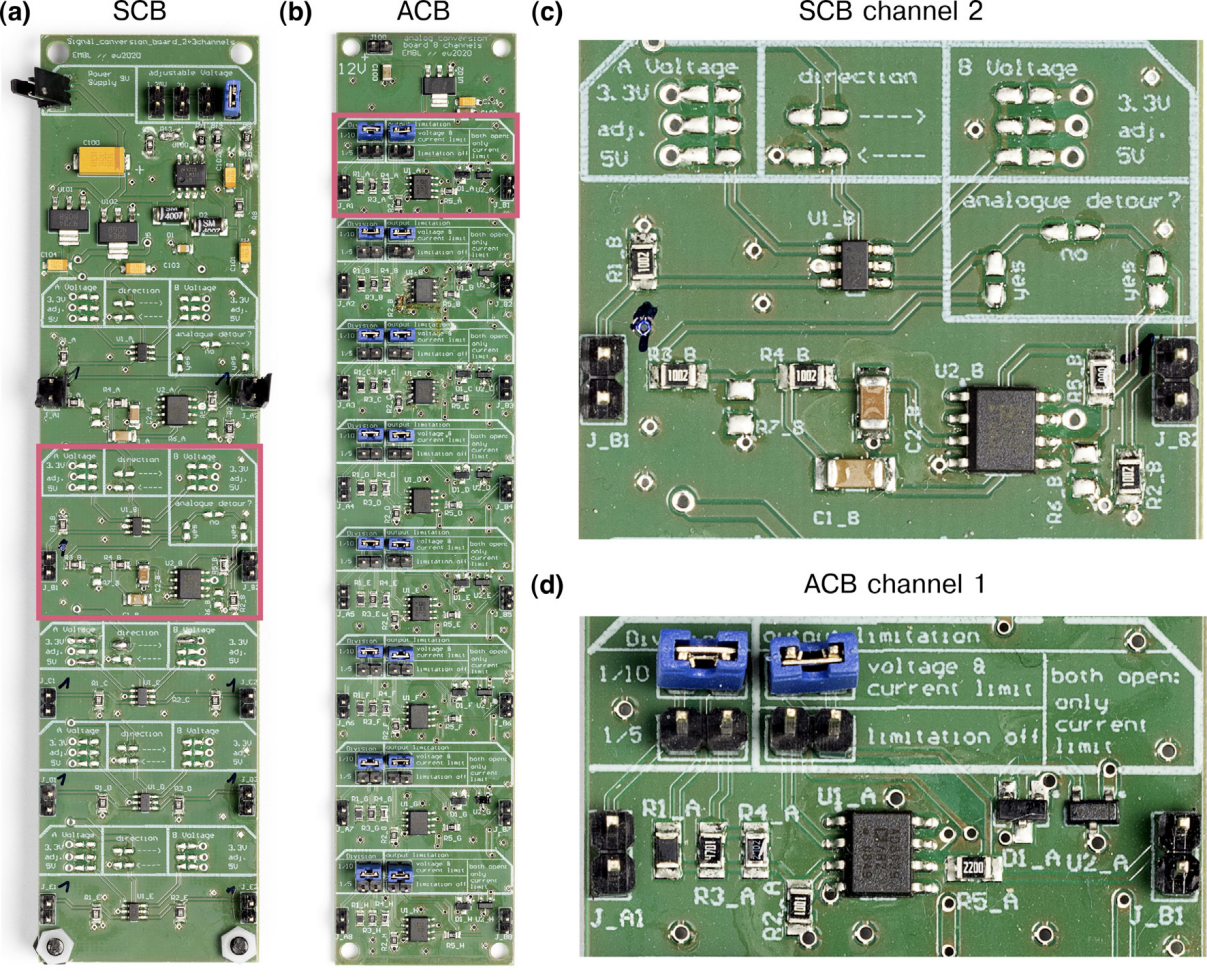
SCB and ACB. (a) Signal conversion board (SCB), with two channels capable of low-pass filtering and three channels with simple voltage scaling. (b) Analog conversion board (ACB). (c) Close-up of the second SCB channel (red rectangle in (a)), with open soldering bridges. (d) Close-up of the first ACB channel (red rectangle in (b)), configured with jumpers. Photography credit: EMBL Photolab – Stuart Ingham.

**Fig. 5:**
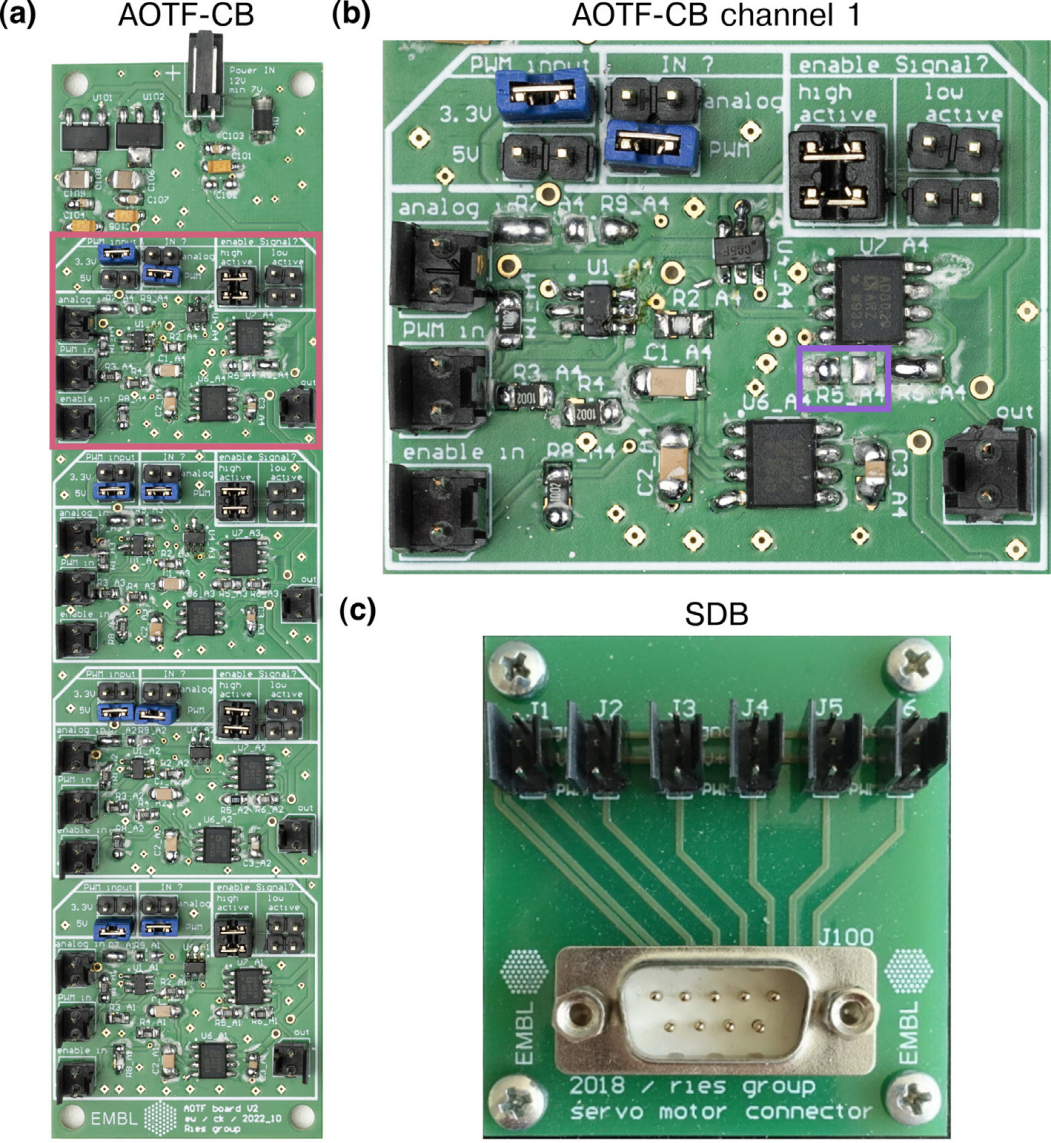
AOTF-CB and SDB. (a) AOTF conversion board (AOTF-CB), with four channels allowing PWM to analog conversion, voltage conversion to the output range and synchronization with a TTL input. (b) Close-up of the first AOTF-CB channel (red rectangle in (a)), configured using jumpers for a 0–3.3 V (“PWM input”) PWM input (“IN?”) with “high active” TTL input (“enable Signal?”). In this channel, we left R5 open (and R6 closed as in the design files) to output a 0–5 V analog signal (magenta rectangle). (c) Servo distribution board (SDB), distributing servomotor signals and power to six different outputs. The D-SUB9 connector should be connected by a flat cable to Box panel 1. Photography credit: EMBL Photolab - Stuart Ingham.

Here, we first describe the complementary electronics (ACB, SCB and AOTF-CB, see Fig. 4 and Fig. 5) and how to test their different channels. We assume that users have followed the steps from **MicroFPGA without optional electronics** related to configuring and controlling MicroFPGA. Finally, we detail how to wire the electronics box following an example used on our microscopes. Note that some of the side panel connectors will not be used and can be adapted to fit the needs of your applications, for instance in the case where additional outputs are needed.

#### 5.5.1 Testing the signal conversion board

The SCB can be used to upscale the FPGA board outputs to 5 V or downscale digital inputs to 3.3 V in 5 independent channels. Selecting the direction and voltage conversion is performed by closing the corresponding solder bridges (see Fig. 4c). It can also be configured to accommodate conversion to an additional custom voltage, other that 3.3 or 5 V. This third voltage level is defined by a set of resistors and needs to be chosen prior to soldering. As some devices are controlled with analog signals rather than digital ones, the SCB also features the possibility to low-pass a PWM signal from MicroFPGA, converting it to an analog signal using a Sallen-Key low-pass Butterworth filter with 10 Hz cut-off (two poles, 10kΩ resistor). Two channels of the SCB have the low-pass filter option (one is shown in Fig. 4c). The resulting analog output voltage range is defined by the aforementioned voltage conversion. Low-passing only functions in one direction on these channels, therefore make sure that the correct left-to-right direction is selected when closing the low-passing solder bridge.

1. Follow the first steps of the **MicroFPGA without optional electronics** section related to configuring and controlling MicroFPGA.
2. Connect a 7 V (1 A) power supply to the  +/- pins at the top of the SCB. You can use box panel 3 for supplying power (see **Wiring the electronics box**, point 7).
3. For each channel, close the solder bridge to select the voltage conversion, the direction of the conversion and whether to use the analog detour (low-pass filter) if applicable (see Fig. 4c). Note that the low-pass filter only works in one direction (left-to-right).
4. For voltage conversion without low-pass filtering, input a voltage from a power supply and check using a voltmeter that the channel output corresponds to the correct conversion. Make sure to respect the direction imposed by the soldered jumper bridge.
5. For voltage conversion of signals generated by the FPGA, you can additionally generate a signal using for instance one of the TTL channels of the FPGA using the software of your choice, connect it to a SCB channel input, and measure the voltage at the channel output.
6. To verify the low-pass filtering, connect an oscilloscope to one PWM output channel of the FPGA. Check the integrity of the signal. Then, connect the PWM output and FPGA GND to the input of one of the two low-pass filter channels on the SCB (J_A1 or J_B1). Use an oscilloscope on the channel output (J_A2 or J_B2) to verify that the PWM signal is converted to an analog signal and that the signal voltage changes with the PWM duty cycle.

#### 5.5.2 Testing the analog conversion board

The ACB converts 0–10 or 0–5 V analog signals to the 0–1 V range. It has 8 uni-directional channels and each of them feature the possibility to limit voltage and current to protect the FPGA (see Fig. 4d). This section is only relevant for the Au+ FPGA.

1. Follow the steps of the first steps of the **MicroFPGA without optional electronics** section related to configuring and controlling MicroFPGA.
2. Connect a 12 V (1 A) power supply to the  +/- pins at the top of the ACB. You can use box panel 3 for supplying power (see **Wiring the electronics box**, point 7).
3. For each channel, select the voltage conversion level by placing a jumper on the division pins (see Fig. 4d).
4. Similarly, select the ”voltage & current limit” using a jumper for each channel (see Fig. 4d).
5. Use a power supply to generate voltage in the range selected by the jumpers and connect the  +/- to the J_A pin.
6. Use an oscilloscope to verify that the voltage conversion works at the channel output. Repeat for all channels of the ACB.
7. Place the Br shield on top of the FPGA.
8. Connect the  +/- of the J_B pins of each channel to the analog input positive and negative pins of the Br shield. Refer to Table 1 of the supplementary data, the documentation [21] or the source code [20] to locate the pins. Alternatively, you can place the custom FPGA shield on top of the Br to simplify testing the analog inputs.
9. Use the software of your choice (Micro-Manager, Python, Java, LabVIEW) to connect to the FPGA.
10. Verify that the FPGA reads out the correct voltages. Note that the FPGA returns a 16 bits value, which needs to be divided by 65 535 in order to obtain the measurement in volts.

#### 5.5.3 Testing the AOTF conversion board

The AOTF-CB (see Fig. 5a) performs a low-pass filtering similar to the SCB, with a Sallen-Key low-pass Butterworth filter with 10 Hz cut-off (two poles, 10kΩ resistor). The board outputs 0–10 V analog signals by design and can perform voltage conversion of the PWM inputs from 3.3 V or 5 V to the output voltage range. By leaving R5 open (and leaving R6 as per the design file) on a channel (exemplified in Fig. 5b, magenta rectangle), the channel output range can be set to 0–5 V. In addition, each channel allows switching on and off the analog output using an input TTL signal (see Fig. 5b). We designed the AOTF-CB to synchronize analog signals with a camera, for instance when controlling laser emission with an AOTF. In such a case, the inputs to an AOTF-CB channel are a PWM signal controlling the laser power and a laser trigger signal synchronized with the camera. The board channels can be used without the TTL synchronization input by selecting the low active option (see Fig. 5b). Finally, it should be noted that the board can also accommodate an analog signal directly instead of a PWM input, while still performing the synchronization with the TTL input. In such a case, the analog input signal should not exceed 5 V.

1. Follow the first steps of the **MicroFPGA without optional electronics** section related to configuring and controlling MicroFPGA.
2. Connect a 12 V (1 A) power supply to the  +/- pins at the top of the AOTF-CB. You can use box panel 3 for supplying power (see **Wiring the electronics box**, point 7).
3. For each channel, select the type of input (analog or PWM), the voltage of the PWM input (if applicable) and the TTL logic (see Fig. 5b). Note that two jumpers are needed to select high or low active TTL.
4. To test the low-pass filtering, you can for instance input a PWM signal generated by the FPGA, without TTL input and with low active logic, and change the PWM level (duty cycle).
5. To test the synchronization, input both a PWM (*PWM in*) and a laser trigger signal (*enable in*) from the FPGA. Configure MicroFPGA to active synchronization mode (no camera input), set reasonable camera parameters and set the laser trigger signal to FOLLOW and sequence 65535. Compare the laser trigger signal with the analog output on an oscilloscope.

#### 5.5.4 Servo distribution board

The SDB (see Fig. 5c) is a hub for the servomotors and should be placed in a central position. It should be connected to Box panel 1 (see Fig. 3b) and distributes signal and power to individual servomotors. The servomotor should be tested together with Box panel 1 (see **Wiring the electronics box**), as it allows easier access to the servo signals.

**Note**: By default, MicroFPGA has 7 servo signals and all of them are mapped to the D-SUB9 pins of Box panel 1 (see next section). However, the SDB has only six servomotor connectors for historical reasons. This means that one servomotor signal is propagated to the SDB but unused. If you need the seventh servo signal, we suggest connecting it to one of the free or unused connectors on one of the side panels.

#### 5.5.5 Wiring the electronics box

In this section, we describe the internal wiring of the electronics box. This example is used on our microscopes and is based on the Au+ FPGA. Since not all channels of the custom FPGA shield and of the side panels will be connected, there is space for connecting additional channels depending on your application.

In this section, we assume that you can connect to a configured MicroFPGA (see the **MicroFPGA without optional electronics** section) and that you have tested the SCB and ACB boards (**Testing the signal conversion board** and **testing the analog conversion board** sections). The testing of the SCB and ACB can also be performed right after point 7 while building the box.

**Note**: If you opted for the Cu FPGA, these instructions remain valid to the exception of the analog inputs. Indeed, the Cu FPGA cannot read analog inputs and therefore the ACB should not be built into the electronics box. The analog pins from the custom FPGA shield and box panel 2 can therefore be repurposed.

1. Based on the requirements of your application, list the type of outputs and inputs, and their signal type and voltage limits. Use this list throughout this step by step guide to adjust the number of channels you need and whether to use SCB or ACB channels.
2. Print the two halves of the box and glue them together. Place the screws and bolts according to Fig. 6.

**Fig. 6:**
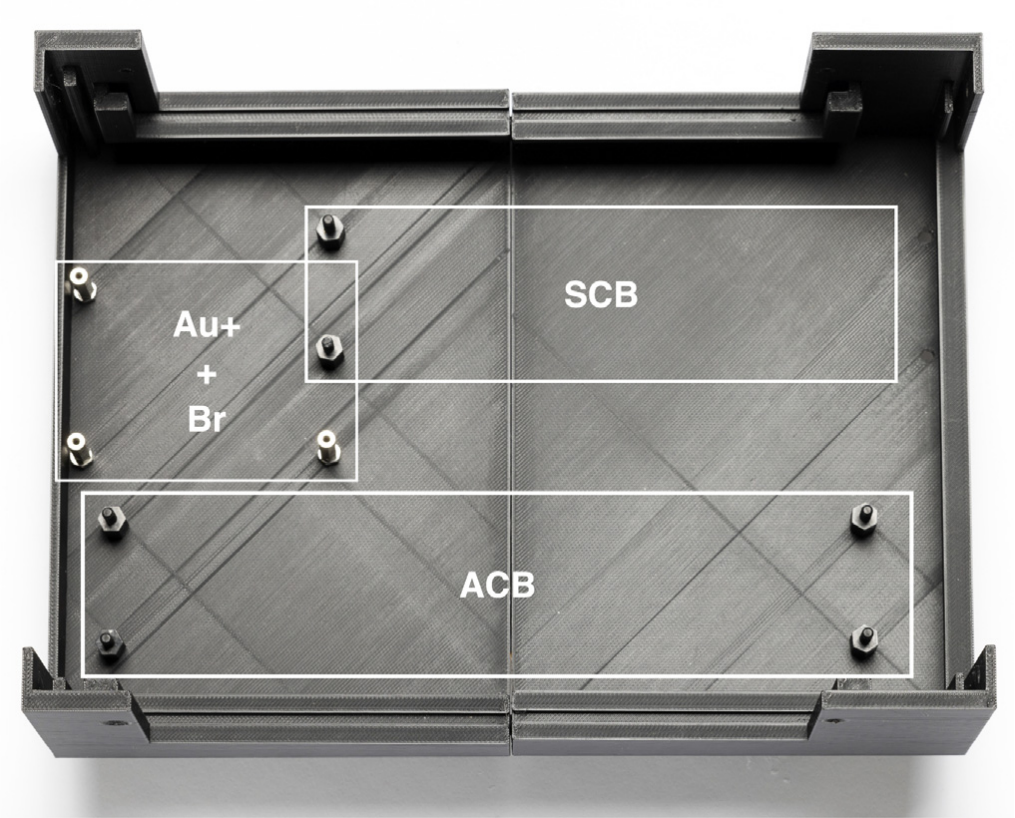
3D printed housing with the boards position outlined. Photography credit: EMBL Photolab – Stuart Ingham.
3. Mount the SCB using the two corresponding screws (see Fig. 6).
4. Mount the Br shield on top of the Au+ FPGA and screw them on the three couplings (see Fig. 6).
5. Then mount the ACB on the remaining screws using nuts (see Fig. 7).

**Fig. 7:**
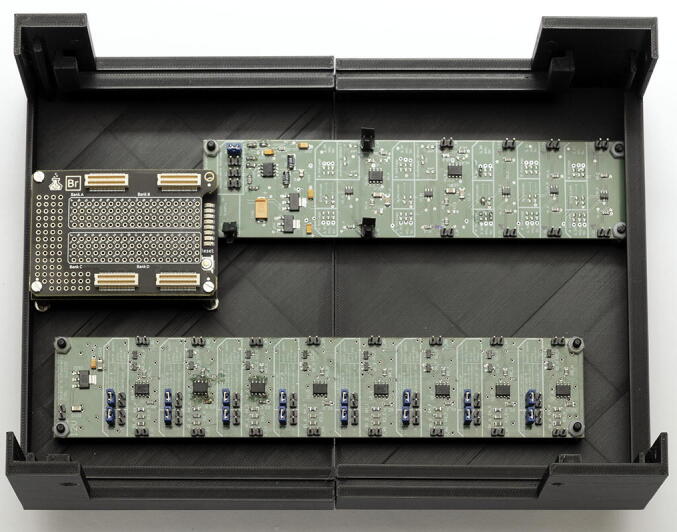
Mounted boards at the bottom of the housing. Photography credit: EMBL Photolab – Stuart Ingham.
6. Mount the custom FPGA shield directly on top of the Br shield. The FPGA shield features connectors that ensure robust connection without possibility to invert GND and signal when plugging in cables.
7. The electronics box’ bill of materials includes pre-fabricated 30 cm cables that are connector crimped on both ends. Since the connection from the power supply connector and P2_12V is short, we recommend cutting a single cable in two. Insert both resulting cables into a connector. Remove the insulation from the free ends and solder them to the back of the power supply plug of Box panel 3 (see Fig. 8). Pay attention to the polarity, as the connector is itself polarized and can only be connected to the 12 V pins (P2_12V) one way. Connect the cables to P2_12V.

**Fig. 8:**
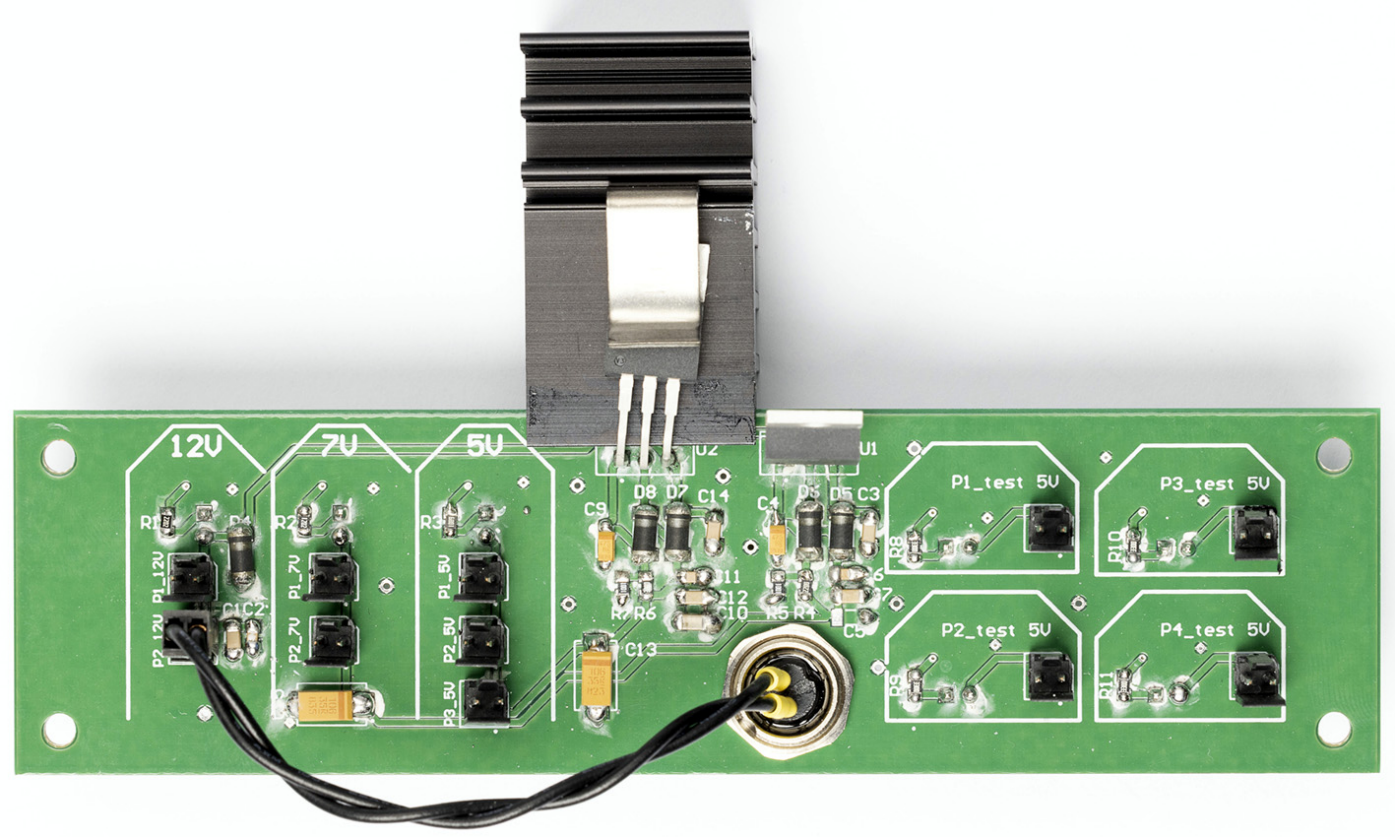
Box panel 3 (voltage source) with the main power source connected to the distribution pins. Photography credit: EMBL Photolab – Stuart Ingham.
  **Note**: Cutting the cables in halves allows saving cables and we use this strategy throughout the electronics box building.
8. Using the pre-made cables, connect one by one the power sources from side panel 3 to the ACB (12 V), SCB (7 V) and custom FPGA shield (5 V), following Fig. 9. We recommend using a lab power supply that shows current and offers current protection instead of the 12 V power supply at this stage. This allows powering each board one at a time and watching the current consumption. Current consumption increasing significantly can be a sign of soldering issues.

**Fig. 9:**
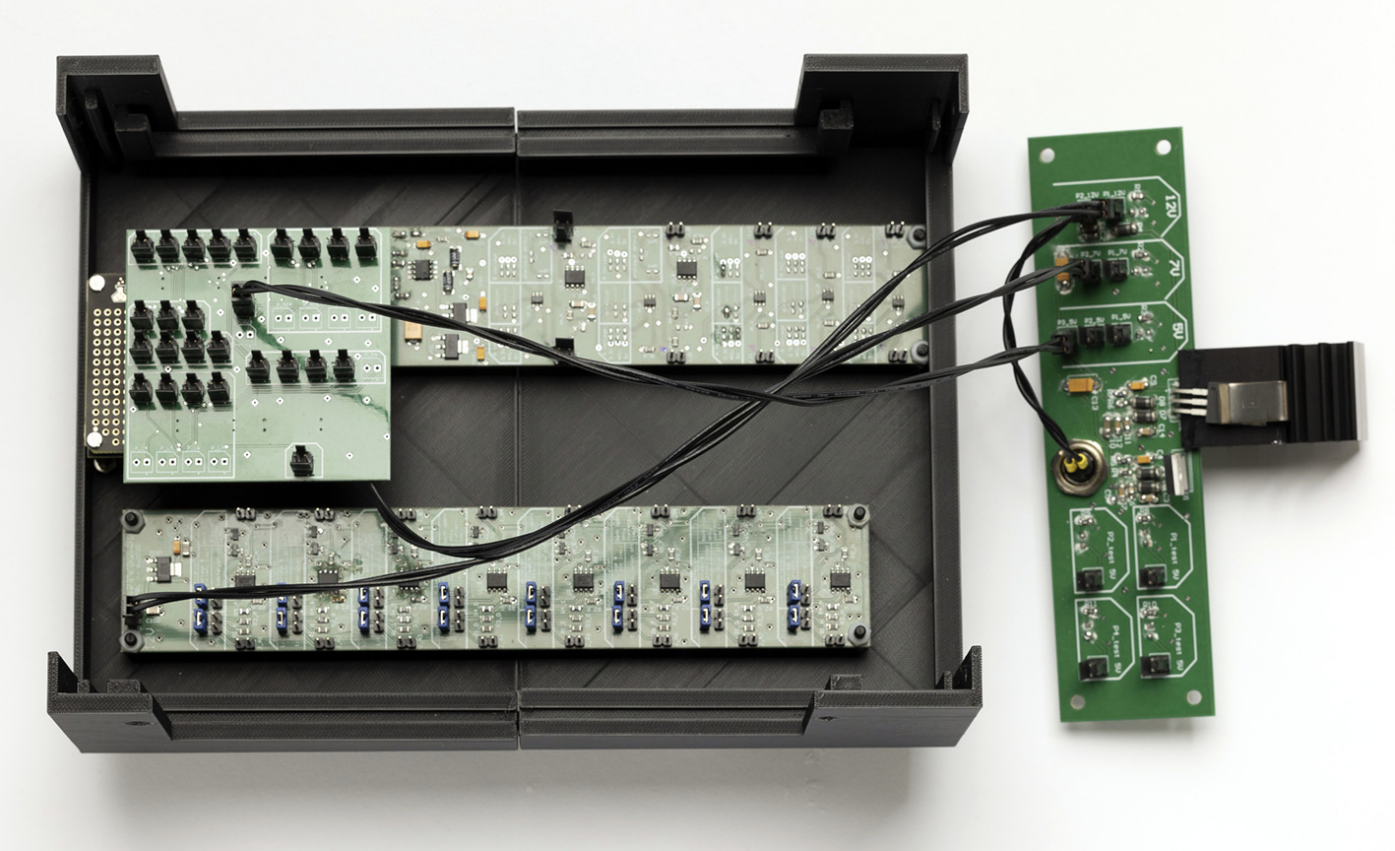
Box panel 3 (voltage source) connected to the ACB, SCB and custom FPGA shield. Photography credit: EMBL Photolab – Stuart Ingham.
9. Wire box panel 2 to the ACB according to Fig. 10: pins J1, J2, and J3 should be connected to the inputs of the first three ACB channels. As they are intended to read signals in the range 0–10 V, place jumpers on the 1/10 voltage conversion and use the voltage and current limit for all channels. The output of each channels are then connected to pins P_AI0, P_AI1 and P_AI2 of the custom FPGA shield. Using a variable power supply, input voltages in the range corresponding to the voltage conversion (0–10 V for the 1/10 conversion) to box panel 2 (channels marked as QPD) and use the software of your choice to compare the FPGA measurement with the input voltage. Remember that the 0–1 V range at the FPGA is measured as a value between 0 and 65 535 (16 bits).

**Fig. 10:**
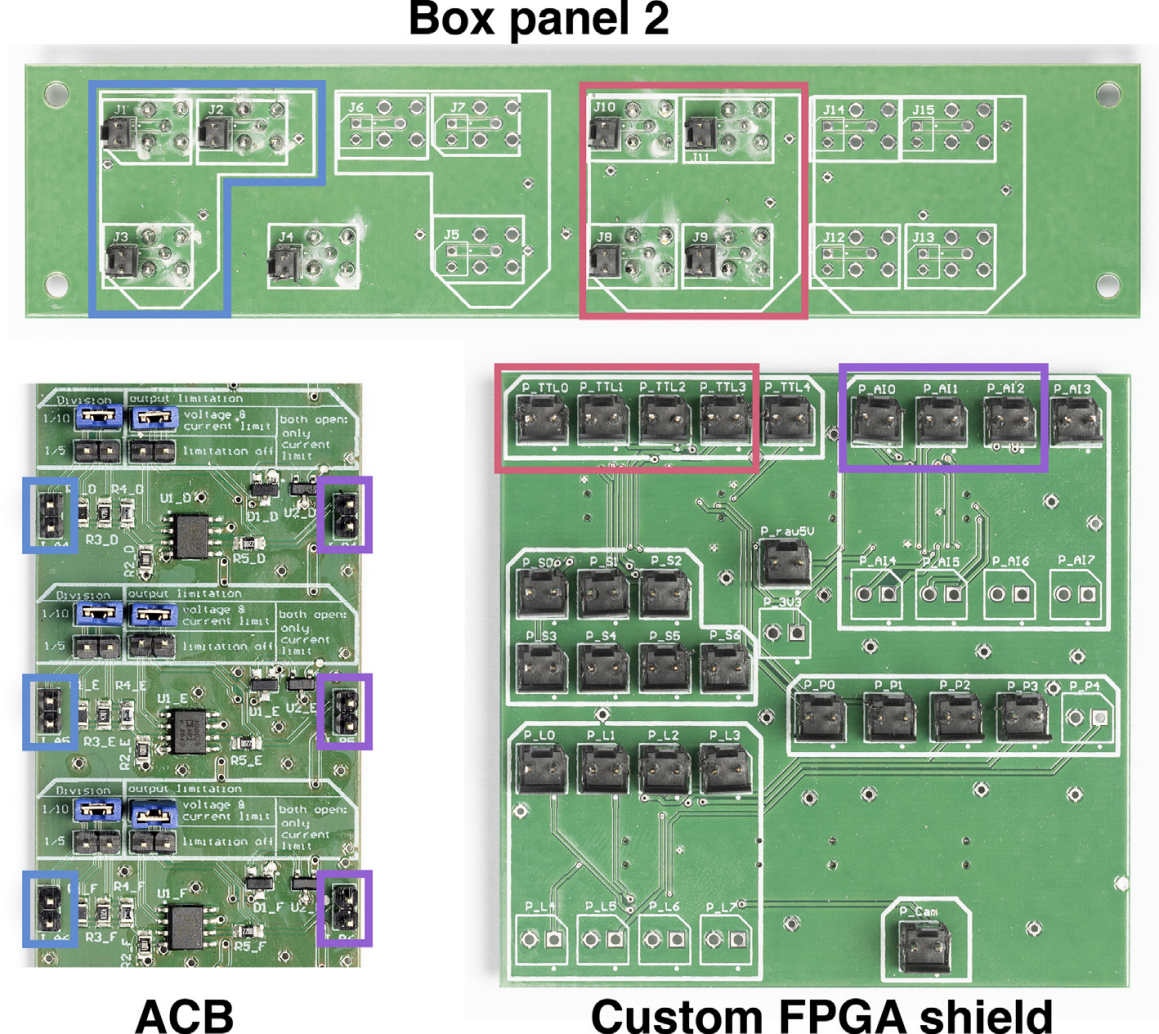
Wiring box panel 2. The TTL outputs from the Custom FPGA shields are connected to the back of box panel 2, pins J8 to J11 (red rectangles), while pins J1 to J3 are connected to the inputs of three ACB channels (blue rectangles). Finally, the outputs of the ACB channels are connected to 3 analog inputs (P_AIx) of the custom FPGA shield (magenta rectangles). Note that for historical reasons the fifth output of the TTL section in the custom FPGA shield is called “P_TTL4”, while it actually corresponds to the camera trigger output (active sync mode, see **Camera synchronization** section). Photography credit: EMBL Photolab – Stuart Ingham.
  **Variation**: If you intend to measure voltages in the range 0–5 V, change the jumpers to 1/5 for the relevant ACB channels.
  **Variation**: If you need more analog input channels, you can use the other pins on box panel 2 (e.g. J4, J5, J6 etc.) and wire them to available channels of the ACB. Then wire the ACB outputs to unused pins P_AI3 to P_AI7 of the custom FPGA shield.
10. Following Fig. 10, wire the TTL pins of the custom FPGA shield to pins J8 to J11 of box panel 2 (red rectangles in Fig. 10). Using the software of your choice, change the state of the TTL channels and confirm the voltage change using an oscilloscope at the exit of box panel 2.
  **Note**: Note that P_TTL4 corresponds to the camera output signal (passive sync mode) and not to a TTL channel.
  **Variation**: If you want the possibility to use MicroFPGA to trigger a camera (active camera mode), then connect P_TTL4 from the custom shield to J14 on box panel 2. Since the output is 3.3 V, it could also be scaled to 5 V via the SCB before connecting to box panel 2.
11. We now move to box panel 1. Pin J17 from box panel 1 corresponds to the camera trigger input (passive sync mode). If the trigger generated by the camera is in the range 0–5 V, then it needs to be scaled down to 3.3 V before reaching the FPGA. In that case, we use a channel of the SCB (preferably one without the optional low-passing) configured to perform 5 V to 3.3 V conversion (left-to-right), as in Fig. 11 (see arrows). Connect pin J17 from box panel 1 to the input of the SCB channel (1, red rectangles, Fig. 11), and the output of the SCB channel to P_Cam on the custom FPGA shield (2, blue rectangles).

**Fig. 11:**
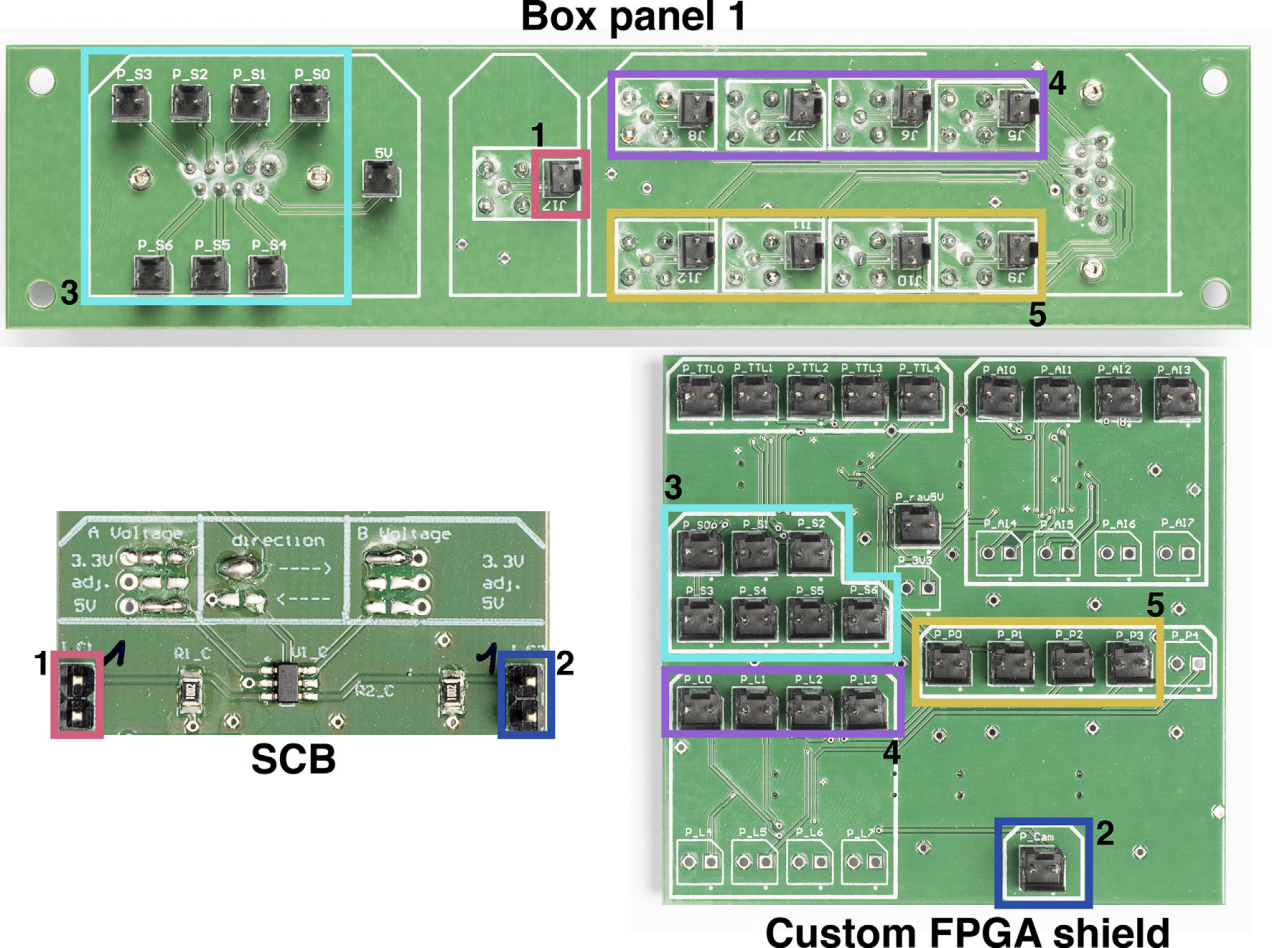
Wiring box panel 1. In this example, the external camera trigger is wired to the input of an SCB channel (1, red). The SCB channel is configured (solder bridges, see arrows) to convert a 5 V signal (1, red) to a 3.3 V output (2, blue). The output of the SCB channel is then connected to the external camera input on the custom FPGA shield (2, blue). The servo signals from the custom FPGA shield (3, cyan) are connected to the corresponding pins on box panel 1, and similarly for the laser triggers (4, magenta) and the PWM outputs (5, yellow). Photography credit: EMBL Photolab - Stuart Ingham.
  **Variation**: If your camera outputs a 3.3 V trigger, as do most (s) CMOS cameras, then you can connect J17 directly to P_Cam.
12. Connect the servo signal outputs P_S0 to P_S6 of the custom FPGA shield to the corresponding pins on box panel 1 (see 3, cyan rectangles, in Fig. 11). Additionally, connect P1_5V of box panel 3 to the 5 V pins of box panel 1. Using the software of your choice, change the state of the servo channels and verify using an oscilloscope that the corresponding box panel 1 outputs change accordingly. You might want to use the SDB for testing, as its pins are more accessible than the panel’s D-SUB9. The SDB should be connected to box panel 1 D-SUB9 servo connector using a D-SUB9 flat cable.
  **Note**: The SDB is intended to be placed at the heart of the microscope, close to the servomotors.
13. Connect the laser trigger outputs P_L0 to P_L3 of the custom shield to pins J5 to J8 of box panel 1 (4, magenta rectangles in Fig. 11). In order to properly test the laser triggering, we need to first input a square TTL signal to the camera input (corresponding to J17) of box panel 1. Make sure to respect the voltage limits (5 V if using the 5 to 3.3 V conversion of the SCB, 3.3 V otherwise). Then using the software of your choice, set the laser channels modes to FOLLOW (mode 4), the sequences to the maximum value (65 535) and the FPGA sync mode to passive.
  **Note**: You can avoid using a square TTL signal in input by setting MicroFPGA synchronization mode to active and the camera parameters exposure and read-out to reasonable values. Then, the FPGA is generating both camera and laser trigger signals.
  **Variation**: If you need more laser trigger outputs, use P_L4 to P_L7 (custom FPGA shield), and connect them to unused PWM channels (J9 to J12) on box panel 1. If all PWM channels are in used, there are still unused channels on box panel 2.
14. Connect the PWM outputs P_P0 to P_P3 of the custom shield to pins J9 to J12 of box panel 1 (5, yellow rectangles in Fig. 11).
  **Variation**: If you need analog outputs, you can use any of the PWM pins from the custom shield and connect them to the left-hand side of one of the SCB low-pass capable channels (Fig. 4c). Make sure that you closed the correct solder bridge: 3.3 V input, left-to-right direction, 5 V output and the two ”yes” in the analog detour (see Fig. 4c). Finally, connect the SCB channel output to the correct connector on box panel 1. Test the box panel 1 PWM outputs using the software of your choice and an oscilloscope.
  **Note**: If you not only need analog outputs in order to control devices, but also want to synchronize them with a camera (active or passive sync mode), you can wire the PWM signal directly to box panel 2. Then, you can use the output PWMs and laser trigger channels as inputs to the AOTF-CB to generate synchronized analog signals.
  **Variation**: If you need more analog signals, the AOTF-CB has PWM to analog channels and can be used without synchronizing TTL inputs.
15. Finally, insert the side panels into the ridges and screw them onto the box.

#### 5.5.6 Connecting to devices

The connection to the devices requires compatible cables on the device side (BNC, SMA, SMB etc.) and female SMB for connecting to the electronics box. The SDB can be connected to the electronics box using a D-SUB9 flat cable. It can then be placed closer to the servomotors. The AOTF-CB has not been intended to be placed inside the box, but could replace the SCB inside the box or fit next to it in a larger box.

## 6 Operation instructions

Since MicroFPGA is aimed at controlling devices on microscopes, operating the FPGA can impact the state of lasers through the laser triggering module. Always make sure that the laser safety precautions are in place when triggering lasers with MicroFPGA. Likewise, when moving elements (stages or servos) with MicroFPGA, make sure to only move them within a safe range that does not endanger fragile microscope components. Finally, make sure that the FPGA and the electronics box are grounded and that the power supply is well soldered and protected from direct contact with metal or touch.

## 7 Validation and characterization

### 7.1 Camera and laser synchronization

**Fig. 12:**
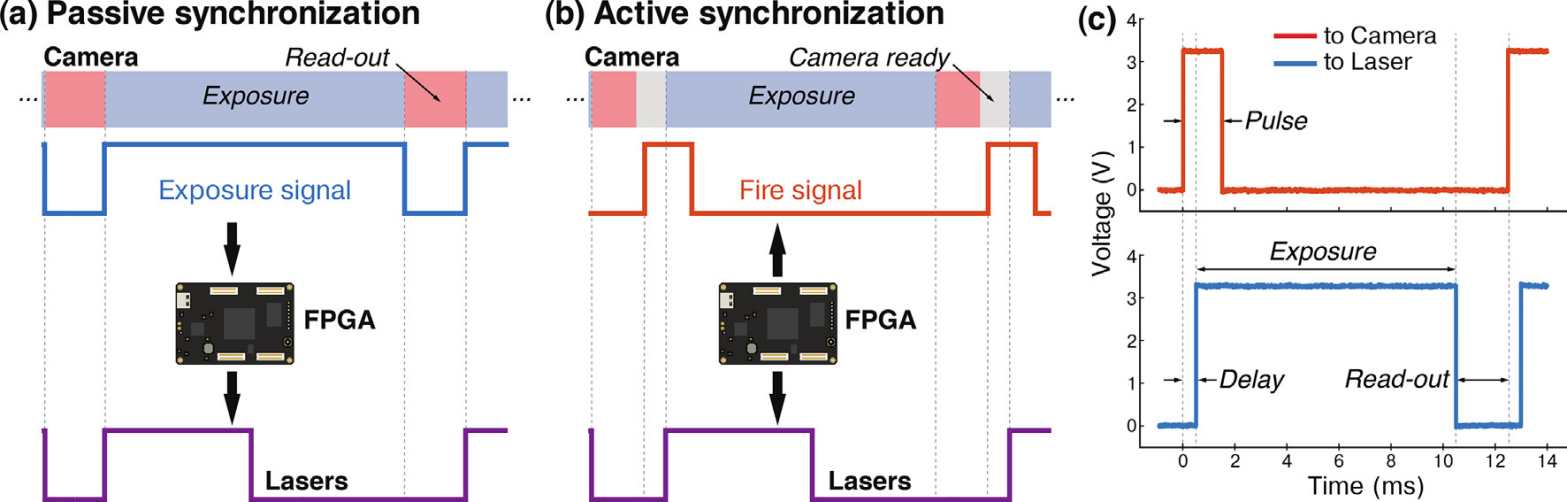
Passive and active synchronization. (a) In passive synchronization, the camera generates an *exposure signal* (blue), which is then processed by the FPGA in order to trigger the lasers (purple). (b) In active synchronization, the FPGA generates a *fire signal* (red) used to trigger the camera. There is usually a delay between the camera receiving a pulse and the start of the next frame acquisition. The FPGA also generates the laser trigger signals (purple), in sync with the *fire signal*. (c) Active synchronization requires four parameters: the pulse parameter is the pulse length of the *fire signal* (in red), the delay parameter introduces a delay between fire and an internal *exposure signal* (see red and blue signals), while the exposure parameter is the pulse length of the *exposure signal* (in blue) and the read-out parameter introduces a pause between the end of the exposure and the next fire pulse. We used the following camera parameters: pulse  = 1.5 ms, delay  = 0.5 ms, exposure  = 10 ms and read-out = 2 ms.

Most cameras used in microscopy, whether (scientific) complementary metal–oxide–semiconductor ((s) CMOS) or electron multiplying charge-coupled device (EMCCD), can be synchronized with other devices using TTL signals. Synchronization between the camera and the lasers avoids for instance unnecessary excitation and bleaching of the sample in between two consecutive frames. The nature of the synchronization depends on the camera type and its trigger modes. For instance, the camera often emits a TTL signal corresponding to the time interval where light is collected by the sensor. In such cases the TTL signal, here referred to as the *exposure signal* (see blue signal in Fig. 12a), is high when the camera is exposing and low when it is registering the sensor pixel values. Alternatively, cameras can be triggered by a TTL input. Each new pulse of the TTL input triggers the acquisition of a new frame. We here refer to the TTL input as the *fire signal* (see red signal in Fig. 12b). In some cases, the exposure time is set directly by users through an acquisition software, or encoded in the duration of each pulse of the *fire signal*.

#### 7.1.1 Camera synchronization

MicroFPGA offers two synchronization modes for the camera: passive and active. In passive sync mode (see Fig. 12a), the camera generates an *exposure signal* that is processed by the FPGA in order to trigger the lasers. Rather than simply relaying the *exposure signal* to the lasers, as typically done, the processing allows more flexibility in the laser behaviour (see the next section for a description of the laser triggering parameters).

In active sync mode, the FPGA generates both the input trigger to the camera (*fire signal*) and the laser trigger signals (see Fig. 12b). In order to cover a large range of camera modes, the *fire signal* is entirely parameterized by four parameters: pulse, delay, exposure and read-out (see Fig. 12c). The pulse parameter corresponds to the pulse length of each pulses in the *fire signal*. The pulse duration can be set to a small value or to the full exposure length, depending on whether the camera expects the *fire signal* to encode the exposure time (refer to the camera user guide for the different triggering modes available for your camera). In active sync mode, the FPGA also generates an internal *exposure signal*. This signal is then processed exactly as an actual camera’s *exposure signal* in passive synchronization mode. Most often in a real setting, there is a delay between the camera receiving a *fire signal* pulse and the start of the exposure. The delay parameter accounts for such a delay between the *fire* and *exposure signal*. After the delay, the *exposure signal* is pulsing for the duration of the exposure parameter. Finally, the next *fire signal* pulse starts after a read-out period, leaving time for the camera to register the pixel values before the next pulse. This means that the *fire signal* is periodic, with a period equal to the sum of the delay, exposure and read-out parameters. All parameters are set in steps of 1 μs with a maximum value of about 1 s for the pulse and exposure parameters, and 65 ms for the other parameters. MicroFPGA waits for a computer command before generating the signals and similarly stops the triggering upon receiving the corresponding instruction (see the extra *start* parameter in Table 1). The precision of the different parameters were measured from a sequence of frames (N = 158), and led to mean ± standard deviations equal to 1499.525 ± 0.0025 μs (pulse  = 1500 μs), 499.79 ± 0.68 μs (delay  = 500 μs), 9999.99 ± 0.16 μs (exposure  = 10000 μs) and 1000.48 ± 0.23 μs (read-out  = 1000 μs).

The various parameters should be chosen in agreement with the specifications and settings of the camera. For instance, for a camera triggered at every pulse and a fixed exposure length set by the acquisition software, the pulse parameter can be experimentally set by increasing the pulse length until frames are received by the computer. The exposure parameter should reflect the exposure from the acquisition software. The delay and read-out parameters depend on the camera itself, and can be approximated experimentally by measuring the pixel intensity in the acquired frames while changing the parameters.

#### 7.1.2 Laser triggering

**Fig. 13:**
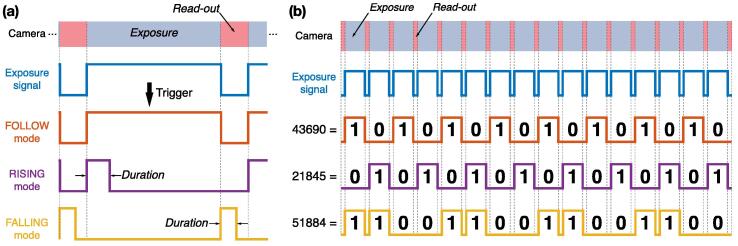
Laser triggering parameters. (a) The camera or the FPGA generate an exposure signal (blue) used to trigger the lasers according to different modes: off (not illustrated here), on (idem), follow (red), rising (purple) and falling (yellow). The duration parameter only affect the rising and falling modes. (b) The sequence parameter affects the triggering modes illustrated in (a). It is a sequence of 16 ones and zeroes, each corresponding to the laser being on (1) or off (0) during a frame within a sequence of 16 frames. The decimal number corresponding to the binary sequence is shown on the left.

**Fig. 14:**
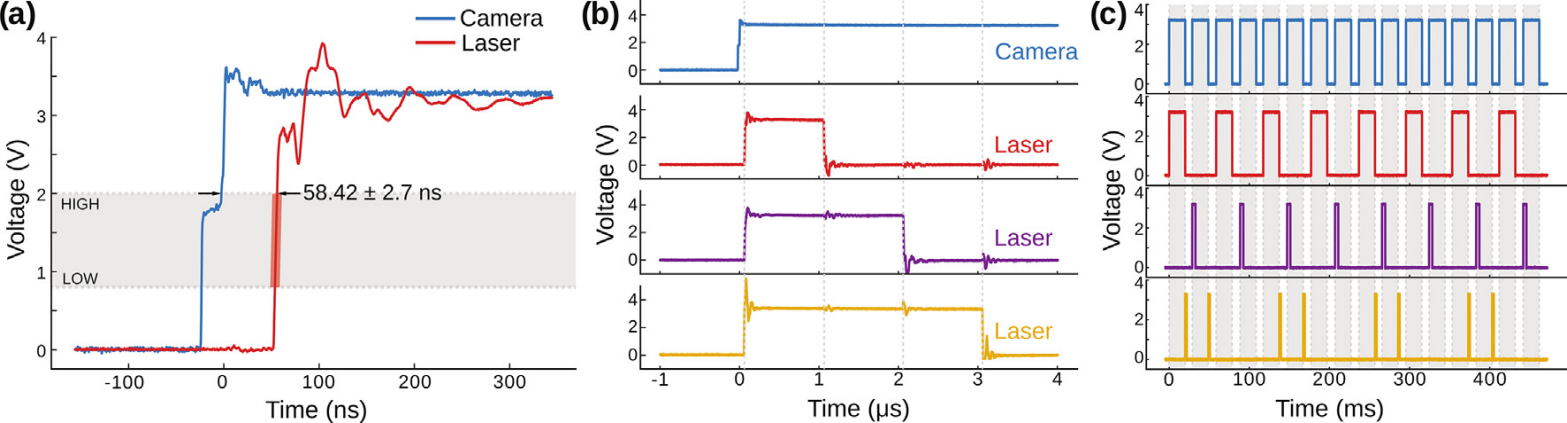
**Laser triggering in passive synchronization.** (a) Measured delay between the camera *exposure signal* (blue) and a laser trigger generated by the FPGA (red, passive synchronization). The laser trigger corresponds to the median delay of 203 signals recorded sequentially. The average delay ± standard deviation is indicated in the plot. LOW (0.8 V) and HIGH (2 V) thresholds are the typical maximum (LOW) and minimum (HIGH) TTL input voltages for the corresponding states. The shading during the transition corresponds to the maximum and minimum observed delays. (b) Multiple lasers can be pulsed in parallel with different pulse lengths, example here with 1 μs (red), 2 μs (purple) and 3 μs (yellow), while the *exposure signal* is shown in blue. (c) Triggering pattern of three lasers using the following parameters: [mode, duration (μs), decimal sequence = binary sequence]. Laser 1 (red) with [follow, not applicable, 43 690 = 1010101010101010], laser 2 (purple) with [rising, 4 000, 21 845 = 0101010101010101] and laser 3 (yellow) with [falling, 2 000, 52 428 = 1100110011001100]. The camera exposure signal is shown in blue.

**Fig. 15:**
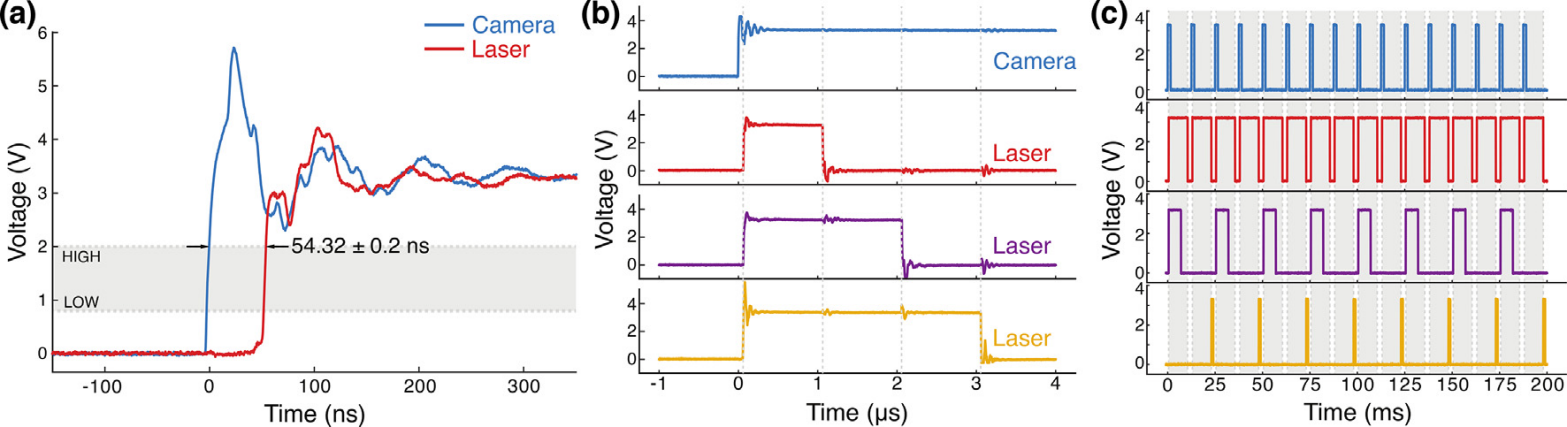
Laser triggering in active synchronization. (a) Measured delay between the *fire signal* (blue) and laser trigger (red), both generated by the FPGA in active synchronization. The laser signal corresponds to the median measured delay of 203 signals recorded sequentially. The average delay ± standard deviation is indicated in the plot. LOW (0.8 V) and HIGH (2 V) thresholds are the typical maximum (LOW) and minimum (HIGH) TTL input voltages for each corresponding state. We did not use any delay (camera parameter). (b) Multiple lasers can be pulsed in parallel with different pulse lengths, here with 1 μs (red), 2 μs (purple) and 3 μs (yellow), while the *fire signal* generated by the FPGA is shown in blue. We did not use any delay. (c) Triggering pattern of three lasers using the following parameters: [mode, duration (μs), decimal sequence  = binary sequence]. Laser 1 (red) with [follow, not applicable, 65 535 = 1111111111111111], laser 2 (purple) with [rising, 6 500, 43 690 = 1010101010101010] and laser 3 (yellow) with [falling, 1 000, 21 845 = 0101010101010101]. The *fire signal* is shown in blue. We used the following camera parameters: pulse  = 1.5 ms, delay  = 0.5 ms, exposure  = 10 ms and read-out = 2 ms.

Regardless of the camera synchronization mode, MicroFPGA processes an (internal or external) *exposure signal* and allows complex triggering of multiple independent lasers (by default 8). Five different trigger modes are available: on, off, rising, falling and follow. In on and off modes, the laser remains in the same state regardless of the *exposure signal*. In rising and falling modes, the laser is pulsed at the rising or falling edge of the *exposure signal* (see Fig. 13a), respectively. The pulse length in both rising and falling modes can be set using the duration parameter. Finally, in follow mode, the laser follows the *exposure signal* (Fig. 13a). When triggering a laser, we observe a mean delay of 58.4 ± 2.7 ns (N = 203) between the camera and the laser signal (see Fig. 14a) in passive synchronization mode, and 54.3 ± 0.2 ns (N = 232) in active mode (see Fig. 15a). The pulse duration in rising and falling modes is between 0 and 65535 μs, with 1 μs steps. Fig. 14b (passive sync) and Fig. 15b (active sync) showcase an example of three lasers simultaneously pulsed for 1, 2 and 3 μs. Since the FPGA has a 100MHz clock and fast rise time, the pulses are extremely accurate, with standard deviations on the order of 0.1 ns (N = 205 and 208 for passive and active sync modes, respectively). If the pulse duration is longer than the triggering interval of the camera corresponding to the exposure, the pulse length is automatically shortened to this interval.

All periodic trigger modes (rising, falling and follow) follow a triggering pattern parametrized by a sequence of 16 bits. Each bit represents a camera frame at which the laser is triggered (1) or not (0) (see Fig. 13b). Since the laser signals share a unique counter, the patterns of all lasers, although independent, are synchronized. Fig. 14c demonstrates the flexibility of MicroFPGA triggering system; the first laser is in follow mode but triggered only every two frames (sequence parameter equal to 43 690 or 1010101010101010 in binary), the second laser is triggered on the rising edge (rising, duration equal to 2 ms) with a pattern opposite to the first laser (sequence equal to 21 845 or 0101010101010101 in binary), and finally, the third laser is pulsed on the falling edge (falling, duration equal to 2 ms) with the following pattern: 1100110011001100 (sequence equal to 52 428). A similar triggering experiment in active synchronization can be found in Fig. 15c.

**Fig. 16:**
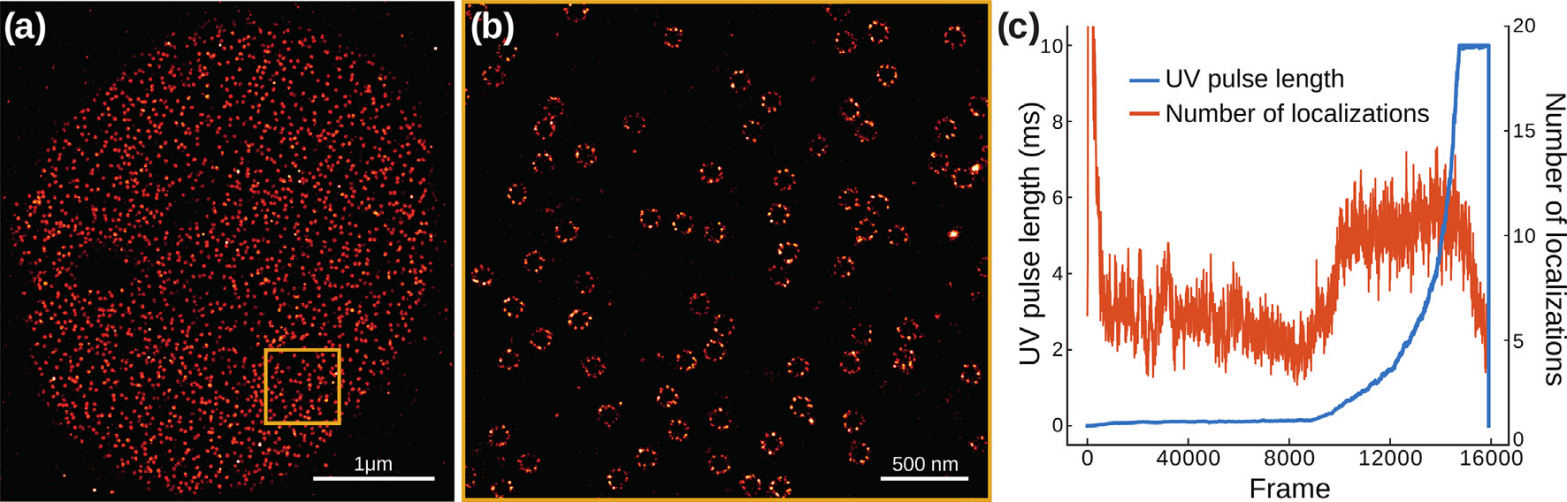
Superresolution imaging. (a) Superresolved image of nuclear pores imaged with single-molecule localization microscopy (160 000 frames) in active synchronization mode. (b) Zoom into the orange region of (a). (c) UV laser pulse length (blue) and number of localizations per frame (red, rolling average of 100 frames) for each frame of the experiment presented in (a).

Such a flexible laser triggering increases the experimental possibilities on the microscope. In particular, the duration parameter allows fine control of the total laser power per frame. For instance, in single-molecule localization microscopy (SMLM) [25], [26], [27] an activation laser (such as a UV laser) is commonly used to randomly activate a subset of the fluorescent molecules in each frame. Because the molecules bleach over the duration of the experiments, the pool of molecules that can still be activated decreases over time. In order to optimize imaging speed, this decrease is compensated by increasing the power of the activation laser throughout the experiment. Instead of manually increasing the laser power over hours, automation of the laser power is an important aspect of an SMLM microscope. Rather than using the raw laser power percentage, MicroFPGA allows using the pulse length of the laser, drastically increasing the dynamic range of the effective power of the activation laser in the sample. We illustrate this in Fig. 16a and b with a superresolved image obtained with an acquisition of 160 000 frames with automated activation. During the experiment, the pulse duration of the activation laser (rising mode) is slowly increased from 0 μs to a user defined maximum of 10 ms (in steps of 1 μs) based on the number of localizations measured at each frame (see Fig. 16c).

**Fig. 17:**
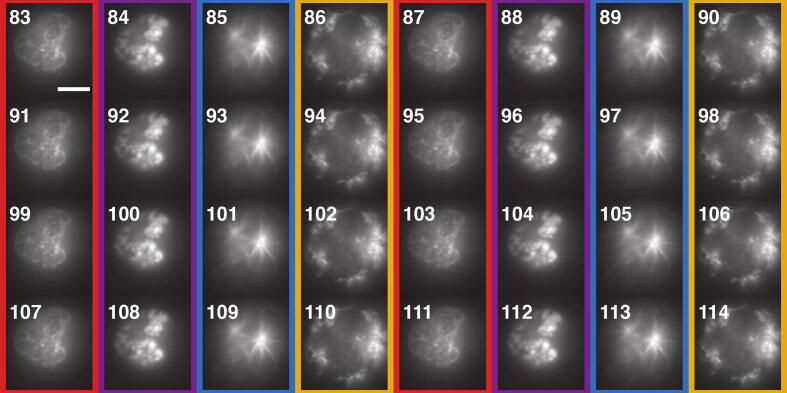
4-color alternating acquisition. Frames 83 to 114 (total of 200) from a 4-color acquisitions. The columns are colour-coded according to the detected structure: Nup96 (red), DNA (purple), Tubulin (blue) and Tom20 (yellow). The four laser lines (405 nm, 488 nm, 561 nm and 640 nm) are alternating using the sequence parameter of the laser triggering module. Scale bar: 10 μm.

The mode and sequence parameters also enable new experiments to be carried out. An obvious application is multi-color imaging by alternating the laser lines during the experiment. For instance, alternating laser wavelengths can reduce cross-talks between channels, or separate in time the different colors when imaging all wavelengths through the same filter. Fig. 17 shows consecutive frames (83 to 114, out of 200) from the same experiment in which four lasers with different wavelengths are alternating. Each laser excites a different fluorescently-labeled structure in the cell: nuclear pores (frame 83), DNA (frame 84), microtubules (frame 85) and mitochondria (frame 86). Fig. 17, visually illustrates the separation in time of the different structures.

### 7.2 PWM and analog output

#### 7.2.1 PWM

PWM is a type of periodic signal that encodes information in the signal duty cycle, that is to say the percentage of time spent in high signal state during a periodic interval. PWM can be used to directly drive certain devices, such as LEDs or servomotors. In addition, low-pass filtering a PWM signal results in an analog signal, which enables controlling devices such as AOTF or galvanometers for instance. By default, MicroFPGA has 5 PWM channels with a period of 1.3 ms and a duty cycle (from 0 to 100%) set using 8 bits (0 to 255).

#### 7.2.2 Analog output

**Fig. 18:**
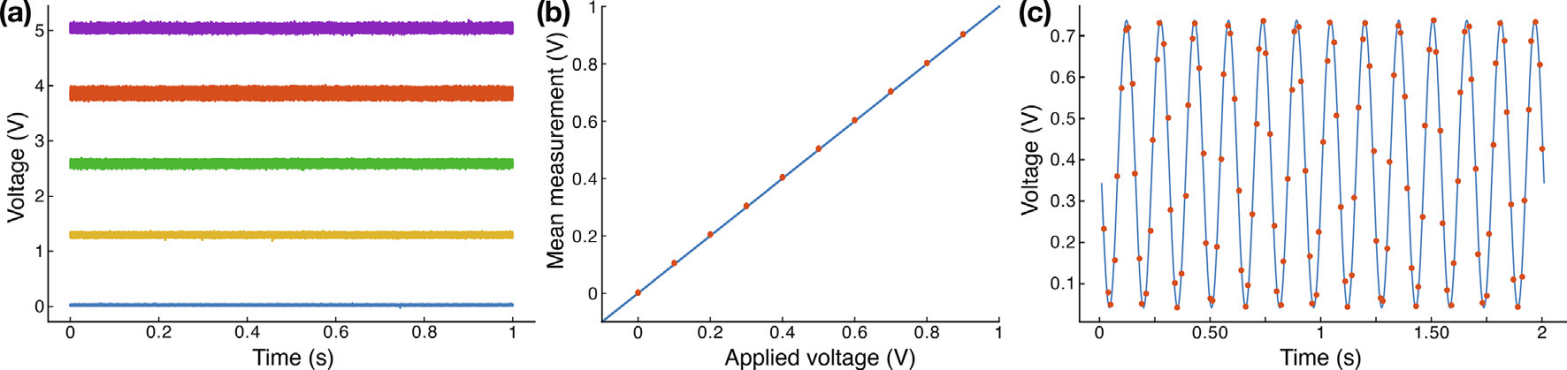
Analog output and input. (a) Analog output voltages measured after passing a PWM signal through the SCB low-pass circuit, corresponding to 0%, 25%, 50%, 75% and 100% of the PWM duty cycle. The average ± standard deviation values are: 0.030 ± 0.005 V, 1.295 ± 0.016 V, 2.578 ± 0.027 V, 3.856 ± 0.034 V, and 5.046 ± 0.028 V, respectively. (b) Average measurement (red dots, 100 samples each) of the analog input signal normalised to the saturation value (65 535) plotted against the applied voltage. The blue line is the expected linear read-out for a perfect analog to digital converter. The standard deviations are comprised between 0.36 and 0.61 mV. (c) Measurements of an analog input channel performed every 16 ms (red dots) and normalised to the maximum value (65 535) as compared to the theoretical input signal (blue line), a sine wave of frequency 6.5 Hz.

When a PWM signal is low-pass filtered, the voltage of the resulting signal is directly proportional to the PWM duty cycle. The period of the PWM channels in MicroFPGA is sufficient to ensure a good low-pass filtering with the SCB board, which requires a PWM faster than 100 Hz and optimally around 1kHz. Fig. 18a shows the analog output resulting from one of the PWM channel, low-pass filtered and converted to 0–5 V range by the SCB. The signal is shown for different values of the duty cycle (0%, 25%, 50%, 75% and 100%). While the noise introduced by the low-pass filter depends on the duty cycle value, it remains fairly small with standard deviations ranging from 0.005 V (at 0%) to 0.034 V (at 75%). For all duty cycle values, the standard deviations are less than 1% of the output voltage. Moreover, the PWM to analog signal conversion is highly linear with only a small offset when fitting a linear curve ($v=1.01\;*\;v_{\mathrm{expected}}+0.05,R=0.99$).

#### 7.2.3 Synchronized analog output

**Fig. 19:**
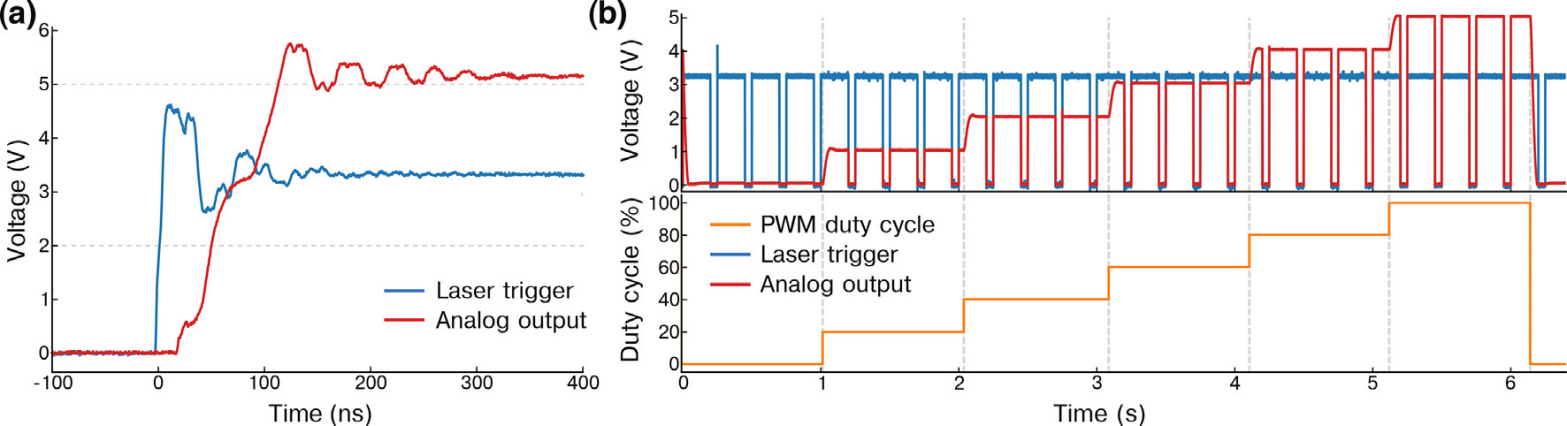
Analog output synchronization (AOTF-CB). (a) Delay between the laser trigger used as TTL input (blue) and an analog output of the AOTF-CB (red). The PWM input to the board was set to 100% duty cycle. The delay measured between the laser trigger signal crossing the TTL threshold (2 V) and the analog output reaching 5 V was 111.70 ± 0.31 ns (N = 197). The reported analog output measurement is the signal corresponding to the median delay. The laser trigger signal was generated using MicroFPGA in active synchronization mode. (b) Example of synchronization between a laser trigger signal used as TTL input (blue, upper panel) and an analog output of the AOTF-CB (red, upper panel), while changing the PWM input duty cycle (yellow, lower panel). The PWM is changed roughly every second to a new value (0%, 20%, 40%, 60%, 80% and 100%). Upon changing the PWM duty cycle (see vertical grey lines), the analog output takes about 100 ms to transition to the new value. The laser trigger switches the analog signal on and off. The camera module was set to active synchronization with the following parameters: [pulse: 225 ms, delay: 0.5 ms, exposure: 225 ms, readout: 24.5 ms], while the laser trigger was set to: [RISING, 200 ms, 65535]. In both panels, the AOTF-CB channel has an output range of 0–5 V.

The AOTF-CB allows multiplying a TTL signal directly with an analog input or with an analog signal generated by low-pass filtering a PWM input. Its low-pass circuit is the same as that of the SCB, a Salley-Key low-pass (two-pole) Butterworth filter with a 10 Hz cut-off. In order to synchronize the analog output signal of an AOTF-CB channel, one can simply use one of the laser trigger signals as TTL input to the channel; regardless of whether the FPGA is in active or passive synchronization mode. As expected from the performances of MicroFPGA’s synchronization, the triggering delay is negligible at time scales usually encountered in microscopy (see Fig. 19a), with an average delay of 111.70 ± 0.31 ns (N = 197). The analog signal is efficiently synchronized with the TTL input, as seen from Fig. 19b (upper panel).

Due to the capacitors used in the Salley-Key low pass circuit (2.25 μF and 1.13 μF), the analog signal value is updated within about 100 ms after the change of the PWM input duty cycle (see Fig. 19b, grey lines). While this is sufficient for setting laser power through an AOTF, it is too slow for fast synchronization such as updating a galvanometer’s position to sweep a laser. For applications requiring high speed synchronization, the AOTF-CB low-pass circuit should be modified using different capacitor values prior to ordering the boards. Finally, the PWM signals frequency should be accordingly increased (see tutorials [21]).

#### 7.2.4 Servomotor control

Servomotors are actuators offering a cost-efficient way to move elements on a microscope. For instance, rotary servomotors can rotate a filter wheel or insert a beam block in the optical path, while linear servomotors can serve as stages. Servomotors are controlled by a particular PWM signal whose duty cycle is limited between a minimum and a maximum value which are different from 0 and 100%. These values correspond to the minimum and maximum servomotor positions. In MicroFPGA, the servomotor signal corresponds to the standard signal, which has a period of 20 ms with pulses between 1 and 2 ms. By default, MicroFPGA offers 7 servomotor channels with 16 bits precision for the position (0 to 65 535). In order to minimize vibrations resulting from servomotor operation on the optical table, the servomotor signals are switched off 10 s after receiving a new position, giving them time to move and settle.

### 7.3 TTL on/off switching

Some hardware devices have the possibility to be switched between two positions (e.g. flip mirrors) or on/off (e.g. lasers) based on an external TTL trigger. We included in MicroFPGA 4 TTL signals that can be toggled between HIGH and LOW states.

### 7.4 Analog input

Finally, the Au+ FPGA includes a 12-bit analog to digital converter (Zynq-7000 SoC XADC, Xilinx), that allows measuring analog signals between 0 and 1 V in multiple channels. Eight such channels are available within MicroFPGA. Fig. 18b demonstrates the high linearity of analog voltage measurements, with standard deviations ranging from $3.6\times 10^{-4}$ V to $6.1\times 10^{-4}$ V. Here, the analog input read-out speed is limited by the serial port communication (RS232) and the transfer between the FPGA and the board microcontroller, the latter being in charge of communication with the computer. To illustrate the board capacity to measure signals in time, we measured a 6.5 Hz sine signal over time (see Fig. 18c). The FPGA board returned a new value on average every 16 ms and the experimental data agreed with the theoretical sine wave (RMSE  = 0.03 V).

### 7.5 Applications

MicroFPGA is a key element of our automated wide-field microscopes and was tested with many different devices (see Table 2). Having a wide range of microscope elements controlled electronically, as it is the case for our microscopes, allows improving the flexibility of the microscope. In particular, the laser triggering allows us to perform localization microscopy experiments with a large dynamic range thanks to microsecond resolution of the activation laser pulses. Since the laser pulse length can be computer-controlled, we implemented an automatic activation script in Micro-Manager [23], allowing for automated localization microscopy. The patterned triggering also allows interleaved excitation between multiple colors, reducing background and enabling more complex experimental designs. We also control multiple servomotors on the microscopes with MicroFPGA (see Table 2), which includes linear servomotors moving 3D lenses, filter wheels and Bertrand lenses, as well as flip-mirrors. During imaging, our microscope can be switched to different channels (by choosing filters), imaging modalities (2D vs 3D) or illumination (TIRF [30] vs homogeneous illumination [31]) without requiring manual user intervention. MicroFPGA was instrumental in developing new imaging techniques [32] or performing high-throughput imaging of biologically-relevant samples [33], [34], illustrating the benefits of microscopy automation.

**Table 2:** Devices tested with MicroFPGA.

**Device type**	**Devices**	**Electronics**	**Signal**
Cameras	Photometrics Evolve 512	SCB	Camera (in)
	Hamamatsu ORCA-Flash4.0 V2	None	Camera (in)
	Hamamatsu ORCA-Quest	None	Camera (in)
	Hamamatsu ORCA-Fusion BT	None	Camera (in)
	PCO edge 4.2 and 4.2bi	None	Camera (in)
	Andor iXon Ultra 897	None	Camera (in)
	Hamamatsu ORCA-Flash4.0 V2	None	Camera (out)
Lasers	Toptica iChrome MLE	None	Laser trigger
	Toptica iBeamSmart	None	Laser trigger
	Omicron LightHUB	None	Laser trigger
	Oxxius LBX-405	None	Laser trigger
	Cobolt Jive 561	None	Laser trigger
	LaserQuantum gem561	None	Laser trigger
	MPB Communications F-04306–107	None	Laser trigger
	MPB Communications F-04–306-102	None	Laser trigger
	LaserEngine [28]	None	Laser trigger and PWM
AOM-AOTFs	Omicron LH.AOM	SCB	Laser trigger and PWM/Analog (out)
	AA Optoelectronic AA.AOTF.6C/TN	AOTF-CB	Laser trigger and PWM/Analog (out)
Servomotors	Sail winch servos [29]	SDB	Servos (out)
Flip-mirrors	Owis KSHM 40	None	TTL
Light sources	LED ring	None	TTL
Sensors	Focus stabilization [29]	ACB	Analog (in)
	Laser power meter [29]	ACB	Analog (in)

### 7.6 Power consumption

MicroFPGA, including the full electronics box, is powered by a 12 V (1 A) power supply. In regular operation in a microscope, we measured currents of about 400 mA. Moving a servomotor leads to short peaks of current. Overall, the current drawn by MicroFPGA is below 1 A, which corresponds to a 12 W power consumption.

## 8 Outlook

In this work, we presented MicroFPGA, an open-source and versatile electronic platform platform that delivers a variety of common signals used to control microscope elements. It is based on affordable FPGAs from Alchitry (Au+ and Cu), and consists of the source code for the FPGA configuration, as well as complementary electronics to convert signals, and libraries to communicate with MicroFPGA from open-source software and languages, Micro-Manager, Java and Python, or the commercial LabVIEW platform.

Further improvements to the current box design could include shielding the MicroFPGA box to improve signal quality. Additionally, MicroFPGA source code is based on RS232 serial communication while the FPGA board is compatible with the open-source USB communication library libusb [35]. USB communication protocol has the potential to deliver faster communication over the FPGA board’s USB port than RS232 currently does. The Java and Python libraries as well as the Micro-Manager device adapter could be modified to use libusb to accelerate communication. USB communication, together with an Ethernet to USB protocol converter, would also enable control of MicroFPGA over a network. Finally, advanced users could also repurpose the code for applications requiring a different type of control, such as synchronizing analog actuators with a camera, galvanometer control, adding another camera input or taking into account camera ready signal in active synchronization.

The configuration code, Micro-Manager, Java, Python and LabVIEW communication libraries, as well as the blueprints for the complementary electronic circuits, are freely available on Github [20] and Zenodo [22], allowing easy integration of MicroFPGA into existing microscopes.

## CRediT authorship contribution statement

**Joran Deschamps:** Conceptualization, Methodology, Software, Validation, Visualization, Investigation, Data curation, Writing - original draft, Writing - review & editing, Supervision. **Christian Kieser:** Methodology, Validation, Investigation. **Philipp Hoess:** Validation, Investigation. **Takahiro Deguchi:** Validation, Investigation. **Jonas Ries:** Conceptualization, Supervision.

## Declaration of Competing Interest

The authors declare that they have no known competing financial interests or personal relationships that could have appeared to influence the work reported in this paper.

## Footnotes

Appendix A Supplementary data associated with this article can be found, in the online version, at https://doi.org/10.1016/j.ohx.2023.e00407.
