# Supplementary material for: MicroFPGA: An affordable FPGA platform for microscope control
Source: HardwareX. 2023 Feb 24;13:e00407. doi: 10.1016/j.ohx.2023.e00407 (PMC9982678; doi:10.1016/j.ohx.2023.e00407)
Supplement: Supplementary file 1 [file mmc1.pdf]

Supporting information

Au+ pin mapping

Note that the Cu FPGA mapping is similar, albeit without the analog inputs.

Signal	Channel	Br pin	Custom shield
Camera input	-	C9	P_Cam
Camera output	-	A5	P_TTL4
Laser	0	C3	P_L0
Laser	1	C2	P_L1
Laser	2	C48	P_L2
Laser	3	C49	P_L3
Laser	4	C46	P_L4
Laser	5	C45	P_L5
Laser	6	C5	P_L6
Laser	7	C6	P_L7
Analog input	0	B24(+)/B23(-)	P_AI0
Analog input	1	B18(+)/B17(-)	P_AI1
Analog input	2	B45(+)/B46(-)	P_AI2
Analog input	3	B48(+)/B49(-)	P_AI3
Analog input	4	B21(+)/B20(-)	P_AI4
Analog input	5	B27(+)/B28(-)	P_AI5
Analog input	6	B6(+)/B5(-)	P_AI6
Analog input	7	B3(+)/B2(-)	P_AI7
PWM	0	A42	P_P0
PWM	1	A41	P_P1
PWM	2	C43	P_P2
PWM	3	C42	P_P3
PWM	4	C8	P_P4
Servo	0	A0	P_S0
Servo	1	A3	P_S1
Servo	2	A49	P_S2
Servo	3	A48	P_S3
Servo	4	A45	P_S4
Servo	5	A46	P_S5
Servo	6	A6	P_S6
TTL	0	A27	P_TTL0
TTL	1	A28	P_TTL1
TTL	2	A9	P_TTL2
TTL	3	A8	P_TTL3

Table 1: MicroFPGA's default pin mapping.

Methods

Programming

The FPGA configuration code was programmed in Lucid within AlchitryLabs 1.2.7 (Alchitry). The Java and Python libraries were developed in IntelliJ 2021.2.3 and PyCharm 2021.2.2 (JetBrains), the Micro-Manager device adapter in Visual Studio 17.0.0 (Microsoft) and the LabVIEW program in LabVIEW 2018 (National

Instruments). The FPGA configurations were compiled in AlchitryLabs with iCEcube2 2017.08 (Lattice Semiconductor, Cu FPGA) and Vivado 2020.2 (Xilinx, Au/Au+), before being flashed to the FPGA using either AlchitryLabs or AlchitryLoader (Alchitry). All source code is available on Github [1].

Electronics

All electronics boards (ACB, SCB, electronics box side panels, Au/Au+ shield) were designed in Altium Designer V22.3.1 (Altium Limited). All Altium projects were exported to Gerber format and the integrity of the projects was verified using gerbv 2.6A. Both SCB and ACB boards were first hand soldered for testing purposes, while the final versions were ordered assembled from a manufacturer (Sigmann Elektronik) with ordering numbers 2020-30170 and reference "Analog Conversion 8 Channels" and "Signal Conversion 2+3 Channels". The Au/Au+ shield and box side panels were ordered with a PCB manufacturer (Q-print electronic GmbH) and the components were hand-soldered. We tested the various boards individually before assembling the box and before wiring each connection. The enclosing box was 3D printed in-house and the transparent plastic lid was made by our mechanical workshop.

Bench measurements

In order to generate Fig. 12c, we connected two channels of an oscilloscope (HDO4054, Teledyne LeCroy) to the camera out (fire signal) pin and the first laser channel of an Au+, through a Br shield (Alchitry). Using a Python script, we set the FPGA to active synchronization and the camera parameters to [pulse (ms), delay (ms), exposure (ms), read-out (ms)] = [1.5, 0.5, 10, 2]. We also set the laser parameters (channel 0) to [mode, duration (µs), sequence] = [follow, N/A, 65 535]. Since the exposure signal is internal in active synchronization, it cannot be measured directly. A laser in follow mode and sequence = 65 535 is the best indirect approximation. We used the oscilloscope to record the two channels for a few tens of milliseconds. Both oscilloscope channels were configured with 1 MΩ impedance.

Laser trigger

Passive synchronization

We used an electronics box featuring an Au board (Alchitry) integrated in a custom microscope, referred here as Microscope A. This microscope has been previously described [2] and includes in particular an EMCCD camera (iXon Ultra 897, Andor) with 3.3 V trigger logic. Because the electronics box is used to control elements on the microscope, it was initially connected to all components (camera, laser, servomotors, filter wheel, bright-field LED ring, flip-mirrors etc.). We added a T-connector on the electronics box to measure the exposure signal generated by the EMCCD camera. Since introducing similar connectors on the laser outputs causes voltages to drop, we disconnected the lasers from the electronics and connected instead the oscilloscope channels. Using Micro-Manager [3], we cropped the camera image to 256×256 pixels, set the exposure time to 20 ms and let the camera run live for the duration of the measurements. We used Python scripts to configure the laser parameters; these scripts are available online [1].

For Fig. 14a and b, the oscilloscope was triggered at 2 V on each rising edge of the camera signal and recorded all channels for a short time window (500 ns and 5 µs, respectively). In Fig. 14a, the laser parameters were [follow, N/A, 65 535]. We recorded 203 samples. We then used another script to detect the time point at which each signal crosses the minimum HIGH TTL value (2 V) and subtracted the difference between laser trigger and camera thresholds to obtain the delays.

In panel b, the three laser parameters were [rising, 1, 65 535], [rising, 2, 65 535] and [rising, 3, 65 535]. As previously, the oscilloscope was triggered on the camera exposure signal and recorded 205 snapshots containing each laser channel. We detected the rising and falling edges for each laser in order to compute the average and standard deviation of their pulse length. We plotted in the figure the median pulse for each laser.

In Fig. 14c, the measurement was run for 69 frames (about 1 s) and the oscilloscope recorded all four channels (camera and three lasers). We extracted a single window of 16 frames for plotting. The laser parameters were

the following: [follow, N/A, 43 690 = 1010101010101010], [rising, 2000, 21 845 = 0101010101010101] and [falling, 2000, 52 428 = 1100110011001100].

Active synchronization

In Fig. 15, the measurement set-up for the active synchronization was the same as that of the passive synchronization, to the exception of the camera signal. Since in this case the FPGA triggers the camera, we simply connected the first channel of the oscilloscope to the FPGA fire signal output. For panel a, the camera parameters were [pulse (ms), delay (ms), exposure (ms), read-out (ms)] = [1.5, 0, 9, 1], and [1.5, 0, 4, 1] in panel b. The laser parameters were the same as those stated in the previous section. The measurement in panel c had the following camera and laser parameters: [1.5, 0.5, 10, 2], [follow, N/A, 43 690 = 1010101010101010], [rising, 4000, 21 845 = 0101010101010101] and [falling, 2000, 52 428 = 1100110011001100]. The analysis pipelines were the same as before.

SMLM experiment

Coverslips were cleaned in a 1:1 mixture of methanol and hydrochloric acid overnight followed by 3 rounds of washing with Milli-Q water and irradiation with UV. U2OS Nup96-SNAP cells [4] were cultivated under adherent conditions in Dulbecco's Modified Eagle Medium (DMEM) supplemented with 10 % [v/v] fetal calf serum, non-essential amino acids, 2 mM L-glutamine (GlutaMAX), and ZellShield at 37 °C, 5 % CO_2, and 100 % humidity. Cells were seeded 2 days before the experiment on clean coverslips with a density to reach 50–70 % confluency on the day of the experiment. All following steps were carried out at room temperature. The samples were pre-fixed in 2.4 % [w/v] formaldehyde (FA) in PBS for 30 s, permeabilised using 0.5 % [v/v] Triton X-100 in PBS for 3 min, and fixed in 2.4 % [w/v] FA in PBS for 30 min on an orbital shaker. Subsequently, they were quenched (100 mM NH_4Cl in PBS) for 5 min, and washed twice in PBS for 5 min each. The samples were blocked with Image-iT FX for 30 min followed by 90 min of staining (1 µM SNAP-Surface Alexa Fluor 647 [NEB], 1 mM DTT, 0.5 % [w/v] BSA, in PBS). Both steps were carried out face down on parafilm in a humidified atmosphere. After the sample was rinsed three times with PBS and washed with PBS three times for 5 min each, the sample was mounted on a custom sample holder in imaging buffer (100 U mL^{-1} glucose oxidase, 0.004 % [w/v] catalase, 10 % [w/v] D-glucose, 35 mM cysteamine, 10 mM NaCl, 50 mM Tris-HCl pH 8).

The experiment was performed on another microscope, Microscope B, described previously [5], but with the camera replaced by a different sCMOS camera (ORCA-Flash4.0 V2, Hamamatsu). Microscope B is also controlled using Micro-Manager and the aforementioned interface. The interface plugin includes both an automated activation script for SMLM and an advanced acquisition module allowing to record superresolution images [6]. The automated activation script subtracts two Gaussian-filtered frames and runs a non-maximum suppression algorithm in order to count the number of localizations live. It then updates the activation laser pulse length based on parameters set by the user, among which an approximate number of localization per frame to aim for.

The camera was set to "Global reset level trigger mode" in order to be triggered by the Au FPGA housed in an electronics box in the microscope. The FPGA was set to active synchronization with the following camera parameters: [28 ms, 0 ms, 28 ms, 2 ms]. The lasers were set to follow mode with sequence equal to 65 535 (all frames), except the activation laser whose mode was set to rising and with an initial duration of 0 µs. We recorded 160 000 frames and stopped the experiment when the pulse duration reached a user defined maximum value.

The raw images were analysed in SMAP [7], using a GPU-accelerated experimental PSF fitting algorithm [8, 9]. The superresolved image was also reconstructed in SMAP. The pulse duration at each frame was extracted from the image metadata, while the number of localizations per frame was exported directly from SMAP [7]. We plotted in Fig. 16c a 100-frame rolling window average of the number of localization per frame to allow interpreting the effect of the automated activation script.

Wide-field experiments

U2OS Nup96-Halo cells [4] were cultivated and seeded as described above. 2 h prior to sample preparation, Halo-JFX646 [10] was added to the medium (f.c. 200 nM). All following steps were carried out on an orbital shaker at room temperature, except for blocking and staining steps which were performed on parafilm in a humidified atmosphere at room temperature. The sample was fixed in 2.4 % [w/v] FA for 30 min, rinsed with 100 mM NH_4Cl in PBS, quenched in 100 mM NH_4Cl in PBS for 10 min, rinsed with 0.25 % [v/v] Triton X-100 in PBS, and permeabilized with 0.25 % [v/v] Triton X-100 in PBS for 30 min. Afterwards, it was washed with PBS for 5 min and incubated in blocking buffer (2 % [w/v] BSA, 0.05 % [v/v] Triton X-100 in PBS) for 30 min. Then, the sample was incubated with the primary antibodies diluted 1:500 in blocking buffer for 2 h: mouse anti-alpha-tubulin (Cat# T6074, Sigma-Aldrich) and rabbit anti-Tom20 (Cat# sc-11415, Santa Cruz Biotechnology). Next, it was washed twice with blocking buffer for 5 min each followed by three rounds of washing with PBS (1, 5 and 10 min, respectively). Afterwards, the sample was incubated with the secondary antibodies diluted in blocking buffer for 1 h: goat anti-mouse Alexa Fluor 488 (1:200; Cat# A-11001, ThermoFisher) and anti-rabbit-CF660C (1:300; Cat# SAB4600310, Sigma-Aldrich). Subsequently, it was washed twice with blocking buffer for 5 min each followed by three rounds of washing with PBS (1, 5 and 10 min, respectively). Next, the sample was post-fixed in 4 % [w/v] FA in PBS for 10 min followed by three washes with PBS for 5 min each. The sample was stored at room temperature overnight. Just before imaging, the sample was stained with DAPI (1:10 000 in PBS) for 5 min followed by three washes with PBS for 5 min each. Finally, the sample was mounted on a custom sample holder in PBS.

We used Microscope A to record sequences of 200 frames with alternating lasers in passive synchronization. In Fig. 17, the lasers parameters were [follow, N/A, 34 952 = 1010101010101010] (DNA, excited by a 405 nm laser), [follow, N/A, 17 476 = 0100010001000100] (Tubulin, 488 nm), [follow, N/A, 8738 = 0010001000100010] (Tom-20, 561 nm) and [follow, N/A, 4369 = 0001000100010001] (Nup96, 640 nm). The montage figures were assembled using Fiji [11].

Analog output

In order to measure analog outputs, we used the oscilloscope directly on the electronics box of Microscope A. We used Micro-Manager to sequentially set the PWM state (channel 0) to 0, 63, 127, 191 and 255. These values correspond to 0 %, ≈ 24.7 %, ≈ 49.8 %, ≈ 74.9 %, and 100 % of the maximum PWM value (255). Within the electronics box, the PWM channel 0 was connected to the first channel of the SCB. This channel converts the signal to the 0–5 V range and low-pass it to obtain an analog output signal. The output of the channel is directly connected to one of the box side panel, allowing to measure the analog output level by plugging the oscilloscope to the box. For each PWM value, we recorded the analog signal for about 1 s. The values are plotted in Fig. 18a. We then averaged the tracks and fitted a linear model using scikit-learn (version 1.2.1) [12].

Analog synchronization

To perform analog synchronization, we used a laser trigger signal and a PWM channel from MicroFPGA as inputs to the first channel of the AOTF-CB. The first channel has an output range of 0–5 V. We then measured both signals and the channel analog output on an oscilloscope while configuring MicroFPGA using Python scripts. Fig. 19a was obtained by triggering the oscilloscope on the laser trigger channel at 2 V, and measuring N=197 traces from the analog signal. The delays were measured as the time difference between the laser trigger crossing 2 V and the AOTF-CB analog output crossing 5 V. The PWM signal was set to its maximum value (255, corresponding to 100 % duty cycle). The camera module was set to active synchronization with the following parameters: [pulse: 1.5 ms, delay: 0 ms, exposure: 9 ms, readout: 1 ms], while the laser trigger channel was set to [mode: FOLLOW, duration: 65 535 ms, sequence: 65535]. In Fig. 19b, we recorded a few seconds of all three signals (laser trigger, PWM and analog output). Using a Python script, we changed the PWM value roughly every second between 0 % (PWM value = 0), 20 % (PWM value = 51), 40 % (PWM value = 102), 60 % (PWM value = 153), 80 % (PWM value = 204) and 100 % (PWM value = 255) duty cycle. The camera module was set to active synchronization with the following parameters: [pulse: 225 ms, delay: 0.5 ms, exposure: 225 ms, readout: 24.5 ms], while the laser trigger channel was set to [mode: RISING, duration:

200 ms, sequence: 65535]. In order to obtain the PWM duty cycle curve, we first detected the rising and falling edge of each pulse, and computed the pulse lengths by subtraction. Since 100 % duty cycle leads to the signal being always on (no PWM pulses), the corresponding pulses had much longer pulse lengths. We replaced these large pulse lengths with the period of the PWM (1.3 ms). Finally, we detected the changes in pulse lengths and averaged piece-wise each segment of constant PWM value. The final curve (Fig. 19b, lower panel) is obtained by plotting the average pulse lengths normalised by the PWM period against the transition times.

Analog input

Fig. 18b was obtained by using a variable power supply (HM8040-3, ROHDE&SCHWARTZ) connected to an analog input channel of an Au FPGA. We manually set the voltage level and used a Micro-Manager script to query 100 measurements and export the results to a file.

Finally, for Fig. 18c, we used a function generator (UTG9005C, UNI-T) to generate a sine wave with frequency 6.5 Hz and amplitude ≈ 8 V. We input the sine signal to an analog input channel of an Au FPGA, and used a Python script to query 100 measurements with a timestamp corresponding to when the query returned. Fig. 18c is obtained by generating a theoretical model of the sine wave using the known frequency and the minimum and maximum measurement values as approximation for the amplitude. We used scipy (version 1.10.0) [13] to optimize the phase of the sine wave on the experimental data. We used a simple Fourier analysis, ignoring the non-uniform sampling, to determine the experimental signal frequency.

References

[1] J. Deschamps, MicroFPGA on Github, https://github.com/mufpga, [Online; accessed 14-February-2023] (2021).

[2] A. Dasgupta, J. Deschamps, U. Matti, U. Hübner, J. Becker, S. Strauss, R. Jungmann, R. Heintzmann, J. Ries, Direct supercritical angle localization microscopy for nanometer 3D superresolution, Nature Communications 12 (1) (2021) 1–9.

[3] A. D. Edelstein, M. A. Tsuchida, N. Amodaj, H. Pinkard, R. D. Vale, N. Stuurman, Advanced methods of microscope control using μManager software, Journal of Biological Methods 1 (2) (2014).

[4] J. V. Thevathasan, M. Kahnwald, K. Cieśliński, P. Hoess, S. K. Peneti, M. Reitberger, D. Heid, K. C. Kasuba, S. J. Hoerner, Y. Li, et al., Nuclear pores as versatile reference standards for quantitative superresolution microscopy, Nature Methods 16 (10) (2019) 1045–1053.

[5] R. Diekmann, M. Kahnwald, A. Schoenit, J. Deschamps, U. Matti, J. Ries, Optimizing imaging speed and excitation intensity for single-molecule localization microscopy, Nature Methods 17 (9) (2020) 909–912.

[6] J. Deschamps, J. Ries, Emu: reconfigurable graphical user interfaces for Micro-Manager, BMC Bioinformatics 21 (1) (2020) 1–13.

[7] J. Ries, SMAP: a modular super-resolution microscopy analysis platform for SMLM data, Nature Methods 17 (9) (2020) 870–872.

[8] Y. Li, M. Mund, P. Hoess, J. Deschamps, U. Matti, B. Nijmeijer, V. J. Sabinina, J. Ellenberg, I. Schoen, J. Ries, Real-time 3D single-molecule localization using experimental point spread functions, Nature Methods 15 (5) (2018) 367–369.

[9] Y. Li, Y.-L. Wu, P. Hoess, M. Mund, J. Ries, Depth-dependent PSF calibration and aberration correction for 3D single-molecule localization, Biomedical Optics Express 10 (6) (2019) 2708–2718.

[10] J. B. Grimm, L. Xie, J. C. Casler, R. Patel, A. N. Tkachuk, N. Falco, H. Choi, J. Lippincott-Schwartz, T. A. Brown, B. S. Glick, et al., A general method to improve fluorophores using deuterated auxochromes, JACS Au 1 (5) (2021) 690–696.

[11] J. Schindelin, I. Arganda-Carreras, E. Frise, V. Kaynig, M. Longair, T. Pietzsch, S. Preibisch, C. Rueden, S. Saalfeld, B. Schmid, et al., Fiji: an open-source platform for biological-image analysis, Nature Methods 9 (7) (2012) 676–682.

[12] F. Pedregosa, G. Varoquaux, A. Gramfort, V. Michel, B. Thirion, O. Grisel, M. Blondel, P. Prettenhofer, R. Weiss, V. Dubourg, et al., Scikit-learn: Machine learning in Python, The Journal of Machine Learning Research 12 (2011) 2825–2830.

[13] P. Virtanen, R. Gommers, T. E. Oliphant, M. Haberland, T. Reddy, D. Cournapeau, E. Burovski, P. Peterson, W. Weckesser, J. Bright, et al., Scipy 1.0: fundamental algorithms for scientific computing in Python, Nature Methods 17 (3) (2020) 261–272.
